# A Ruthenium–BODIPY Photosensitizer for Light-Triggered Apoptosis in Triple-Negative Breast Cancer Cells

**DOI:** 10.1021/acs.inorgchem.6c01479

**Published:** 2026-06-26

**Authors:** Ting-Hsuan Wang, Chia-Hsuan Lin, Eng Zhi Sim, Melody Cai-Syaun Wu, Jack Hau-Ting Wei, Wan Tien Huang, Ricky Yu-Syun Fan, Siao-Cian Pan, Sangeeta, Rakesh Ganguly, Kien Voon Kong, Tiow-Gan Ong

**Affiliations:** † School of Science and Engineering, The Chinese University of Hong Kong, Shenzhen, Guangdong 518172, China; ‡ Department of Chemistry, 33561 National Taiwan University, No. 1, Sec. 4, Roosevelt Rd., Taipei 10617, Taiwan (R.O.C.); § Immune Research Core, Department of Medical Research, National Taiwan University Hospital, No. 7, Chung Shan S. Rd., Zhongzheng Dist., Taipei City 100225, Taiwan (R.O.C.); ∥ Shiv Nadar Institute of Eminence, Delhi-NCR, Greater Noida, Uttar Pradesh 201314, India; ⊥ Center For Emerging Material and Advanced Devices, 33561 National Taiwan University, Taipei 10617, Taiwan

## Abstract

The selective activation of therapeutic agents within the tumor microenvironment remains a cornerstone in enhancing the efficacy and safety of cancer treatments. This study introduces **RuB3**, an agent for photodynamic therapy (PDT), and explores its potential to selectively induce apoptosis in MDA-MB-231 breast cancer cells through controlled light exposure. Employing advanced live cell imaging techniques, fluorescence microscopy, and flow cytometry, we demonstrated that **RuB3** facilitates targeted ROS generation and consequent apoptotic pathways upon light activation. Notably, **RuB3** exhibited a substantial increase in apoptotic effects in a light-dependent manner, confirming its functionality as a PDT agent. Given its potent efficacy and minimal off-target effects, **RuB3** represents a promising advancement in PDT approaches.

## Graphical abstract

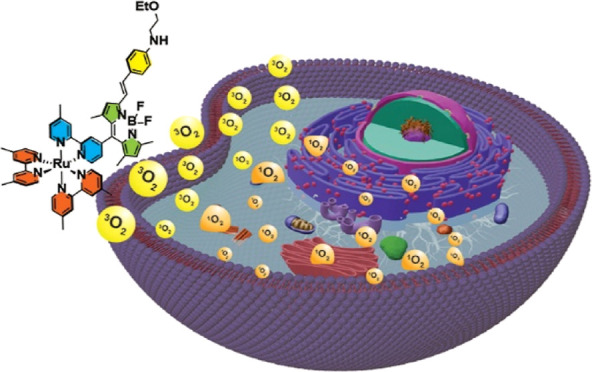

## Introduction

Photodynamic therapy (PDT) is an established anticancer treatment that utilizes a photosensitizer, light, and oxygen to induce photochemically mediated cell death.^1^ Upon irradiation, the photosensitizer is excited to a singlet state and may undergo intersystem crossing to a triplet excited state, which then participates in two principal photochemical pathways. In the Type II pathway, energy transfer to ground-state oxygen yields singlet oxygen (^1^O_2_), whereas in the Type I pathway, electron- or hydrogen-transfer reactions generate radical species such as superoxide and hydroxyl radicals.^2^

Triple-negative breast cancer (TNBC) is a challenging therapeutic target due to its aggressive nature and limited response to conventional treatments.^3^ TNBC cells lack the expression of estrogen, progesterone, and ERBB2 receptors, making them highly invasive and resistant to many therapeutic approaches.^3^ Photodynamic therapy (PDT) has shown promise in combating TNBC by enhancing the sensitivity of multidrug-resistant breast cancer cells to chemotherapeutics.^4,5^ Mitochondria play a pivotal role in cancer cell metabolism, proliferation, and survival.^6^ Researchers have achieved significant inhibition of TNBC growth in preclinical models with PDT.^7–10^

Ruthenium complexes have gained attention in recent years due to their robust photophysical and photochemical properties. Particularly, ruthenium complexes that incorporate polypyridyl ligands have shown potential due to their strong absorption in the visible range, tunable luminescent properties, and ability to act as effective singlet oxygen sensitizers.^11,12^

BODIPY-based chromophores are attractive for PDT because they possess strong and tunable visible-light absorption and high molar absorptivity. In particular, 3-extended BODIPY derivatives exhibit red-shifted absorption and enhanced light-harvesting ability due to their expanded π-conjugation.^13–15^ Such red-shifted absorption is attractive for photodynamic therapy because excitation at longer wavelengths, particularly toward the near-infrared (NIR) region, can enable deeper tissue penetration, reduced light scattering, and improved treatment of deep-seated tumors.^16,17^ Coordination of BODIPY-containing ligands to ruthenium complexes can further improve their photophysical behavior by introducing metal-to-ligand charge-transfer (MLCT) transitions, broadening the absorption window, and modulating the excited-state electronic structure.

In this study, we report the synthesis, characterization, and biological evaluation of a ruthenium-bipyridyl–BODIPY complex, **RuB3** (Figure 1a). Our findings suggest that **RuB3** demonstrates significant potential as a photosensitizer (Figure 1b). Although Ru–BODIPY conjugates have been widely investigated as photosensitizers, most reported systems are based on Ru–arene, Ru–terpyridine, or biotin-tagged Ru–BODIPY architectures and primarily emphasize photophysical properties, singlet oxygen generation, or general photocytotoxicity (Table S1).^18–23^ The novelty of **RuB3** lies not in the general Ru–BODIPY motif, but in its mechanistic demonstration of mitochondria-associated, light-triggered apoptosis in TNBC cells. Consequently, **RuB3** triggers targeted mitochondrial disruption at low nanomolar concentrations, offering a highly precise photodynamic approach that overcomes the targeting limitations of earlier conjugates.

**Figure 1:**
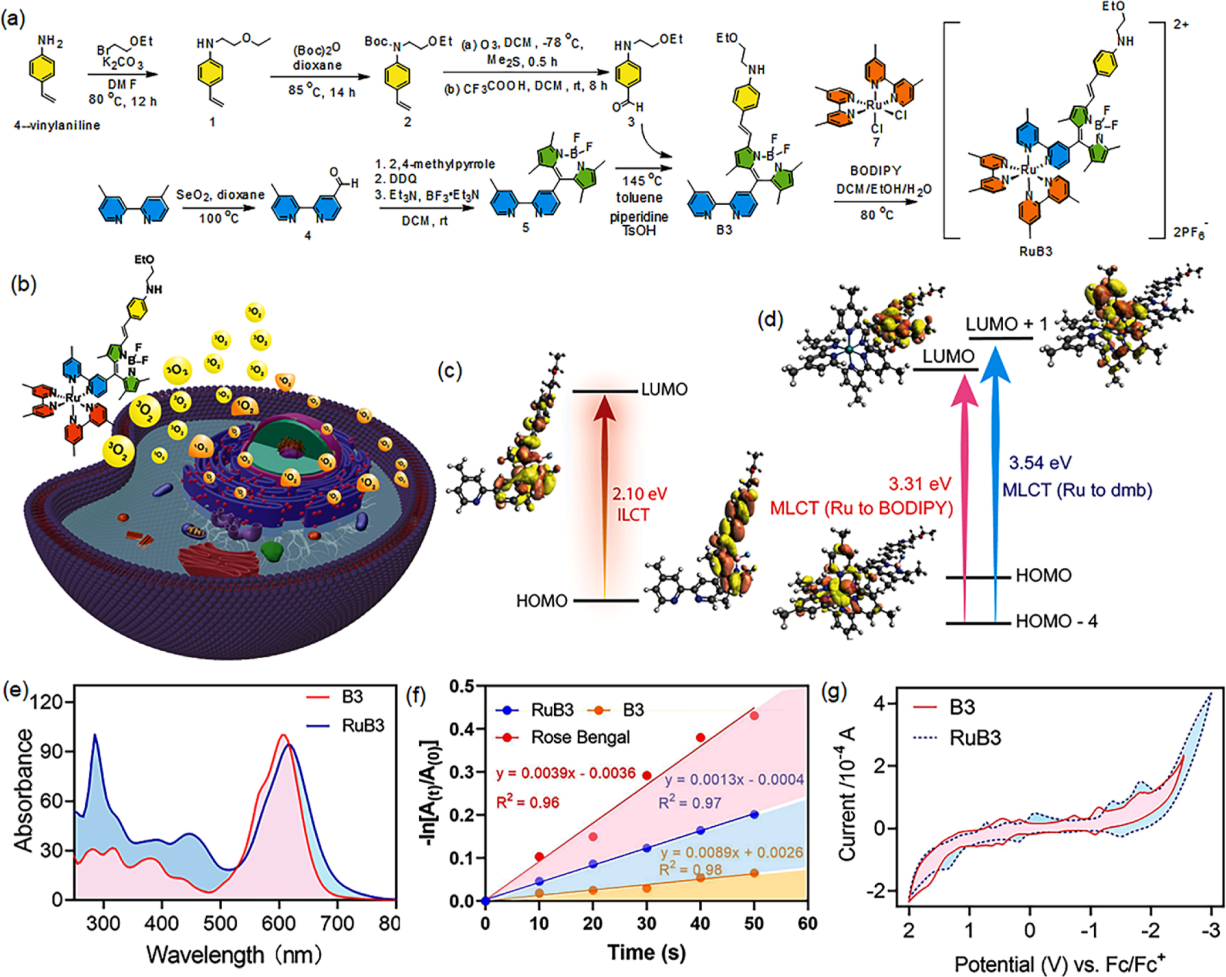
(a) Synthetic scheme of the Ru–BODIPY complex (**RuB3**). The key steps involve the functionalization of BODIPY and its coordination to a ruthenium bipyridine complex. (b) Schematic representation of the intracellular localization of **RuB3** and its generation of reactive oxygen species (ROS) upon light activation. (c) Energy diagram depicting the intraligand charge transfer (ILCT, 2.10 eV) within the BODIPY moiety. (d) Molecular orbitals and electronic transitions of RuB3, highlighting metal-to-ligand charge transfer (MLCT) transitions: Ru to BODIPY (3.31 eV) and Ru to dmb (3.54 eV). (e) UV–vis absorption spectra of B3 and **RuB3**, showing enhanced absorption in the visible region. (f) Singlet oxygen generation efficiency of **RuB3** compared to Rose Bengal and B3, evaluated by monitoring the degradation of 1,3-diphenylisobenzofuran (DPBF). (g) Cyclic voltammograms of B3 and **RuB3**, demonstrating redox behavior in an electrochemical environment.

## Materials and Methods

### Materials

All solvents were dried with the molecular sieves packed solvent purification system and stored in the sealed containers with the 3 Å molecular sieves. 4-vinylyaniline, 2,4-dimethylpyrrole, 4,4′-dimethyl-2,2′-bipyridine (dmb) and RuCl_3_·3H_2_O were purchased from the Nova-Matls. Glucose, ammonium hexafluorophosphate (NH_4_PF_6_) and triehylamine (Et_3_N) were purchased from Sigma-Aldrich. l-ascorbic acid, dimethyl sulfide (Me_2_S), selenium dioxide (SeO_2_), piperidine, 1,4-dioxane and 1,2-dibromoethane were sourced from Acros. Boron trifluoride-diethyl etherate (BF_3_·Et_2_O) and 2,3-dichloro-5,6-dicyano-1,4-benzoquinone (DDQ) were purchased from Alfa Aesar. Potassium carbonate (K_2_CO_3_) and trifluoroacetic acid (TFA) were purchased from the Fisher Scientific. Various kits and dyes, such as Live/dead viability/cytotoxicity kit, Alexa Fluor 488 Annexin V/dead cell apoptosis kit, RedoxSensor Red CC-1, MitoTracker Green FM and LysoTracker Red DND-99 were purchased from the Invitrogen (USA). BioTracker Si-DMA Singlet Oxygen Live Cell Dye was purchased from Merck. Dulbecco Modified Eagle Medium (DMEM), fetal bovine serum (FBS) and penicillin/streptomycin were bought from Gibco.

### Instruments

The NMR spectra were measured on a Bruker AVIII HD 400 MHz spectrophotometer, using tetramethylsilane (TMS) as an internal standard. The mass spectrum was measured by Bruker microTOF-QII (ESI-MS) or Bruker Daltonics Autoflex Speed (MALDI-TOF/TOF) recorded in **m**ethanol. ATR spectrometry was performed with a PerkinElmer FT-IR Spectrometer. The luminescence spectrum was measured by Edinburgh FLS1000 Photoluminescence Spectrometer. UV–vis–NIR spectrum was recorded in the range of 200 to 800 nm with ChromTech CT-2200 spectrophotometer at room temperature. In vitro fluorescence imaging was done by using BioTek Cytation 5 Cell Imaging Multi-Mode Reader and Zeiss Axio Imager Z1 with a 63× oil objective lens.

## Experimental Methods

The procedure for singlet oxygen quantum yield analysis, cell viability assay, intracellular reactive oxygen species, detection, cell apoptosis assay, subcellular localization studies, intracellular superoxide detection, intracellular hydroxyl-radical-associated ROS detection, measurement of mitochondrial respiration, live cell imaging, Western blot analysis, photocytotoxicity under normoxic and hypoxic conditions are provided in the Supporting Information.

### Synthesis of *N*-(2-Ethoxyethyl)-4-vinylaniline (**1**)

The compound was synthesis according to a modified method from the previously published literature. A suspension of 4-vinylaniline (650 mg, 5.45 mmol), 1-bromo-2-ethoxyethane (1.08 g, 7.09 mmol), K_2_CO_3_ (1.506 g, 10.9 mmol) in 40 mL DMF was stirred at 80 °C for 12 h. The reaction mixture was then cooled to room temperature and extracted with 100 mL of DCM three times. The organic layers were combined and washed with water, then dried over MgSO_4_. The filtrate was evaporated to yield a crude product, which was further purified by silica gel chromatography to obtain compound **1** as a yellow liquid. Yield: 32%. ^1^H NMR (400 MHz, CDCl_3_, 298 K): δ = 7.25 (d, *J* = 7.2 Hz, 2H), 6.61 (dd, *J* = 17.1, 10.8 Hz, 3H), 5.52 (d, *J* = 17.7 Hz, 1H), 5.01 (d, *J* = 10.8 Hz, 1H), 4.11 (s, 1H), 3.64 (t, *J* = 5.2 Hz, 2H), 3.53 (q, *J* = 7.0 Hz, 2H), 3.30 (t, *J* = 5.2 Hz, 2H), 1.22 (t, *J* = 7.0 Hz, 3H) ppm. ESI-TOF Mass (*m*/*z*) in methanol (*m*/*z*): calcd, 192.1383 [M + H]^+^; found, 192.1384 [M + H]^+^.

### Synthesis of *tert*-Butyl-2-ethoxyethyl­(5-vinylphenyl)­carbarmate (**2**)

The (Boc)_2_O (412.44 mg, 1.88 mmol) was first dissolved in 1,4-dioxane (5 mL), and then added to a dioxane solution (5 mL) of **1** (328.6 mg, 1.71 mmol). The mixture was stirred under N_2_ atmosphere at 85 °C for 14 h. The solution was rotary evaporated to get a viscous residue, which was then passed through a column chromatography (ethyl acetate/hexane, 1/20, v/v) to afford compound **2** as a brown yellow oil. Yield: 80%. ^1^H NMR (400 MHz, CDCl_3_, 298 K): δ = 7.36 (d, *J* = 8.1 Hz, 2H), 7.21 (d, *J* = 7.9 Hz, 2H), 6.69 (dd, *J* = 17.5, 10.9 Hz, 1H), 5.71 (d, *J* = 17.6 Hz, 1H), 5.23 (d, *J* = 10.8 Hz, 1H), 3.78 (t, *J* = 6.1 Hz, 2H), 3.56 (t, *J* = 6.2 Hz, 2H), 3.46 (q, *J* = 7.0 Hz, 2H), 1.44 (s, 9H), 1.16 (t, *J* = 6.9 Hz, 3H) ppm. ESI-TOF Mass (*m*/*z*) in methanol (*m*/*z*): calcd, 292.1907 [M + H]^+^; found, 292.1901 [M + H]^+^.

### Synthesis of 4-(2-Ethoxyethylamino)­benzaldehyde (**3**)

Compound **2** (280 mg, 0.96 mmol) was dissolved in 10 mL dry dichloromethane, then bubbled ozone (flow rate: 1, 30%) into the solution until it turned into light blue at −78 °C, and ran for further 10 min. Subsequently, Me_2_S (0.3 mL, 0.7 mmol) was added, and the solution was slowly warmed to room temperature within 30 min. A mixture of DCM and trifluororacetic acid in a 1:2 ratio (1.5 mL) was added, and stirred at room temperature for 8 h. The resulting reaction was quenched with 1 M aqueous NaHCO_3_, and then the organic layer was dried over MgSO_4,_ and concentered in vacuum to yield the crude product. The residue was purified by column chromatography with EtOAc/Hex (1:4) to obtain the compound **3** as brown oil. Yield: 59%. ^1^H NMR (400 MHz, CDCl_3_, 298 K): δ = 9.73 (s, 1H), 7.70 (d, *J* = 8.5 Hz, 2H), 6.63 (d, *J* = 8.3 Hz, 2H), 3.66 (t, *J* = 5.2 Hz, 2H), 3.55 (q, *J* = 7.1 Hz, 2H), 3.38 (t, *J* = 5.2 Hz, 2H), 1.24 (t, *J* = 7.0 Hz, 3H) ppm. ESI-TOF Mass (*m*/*z*) in methanol (*m*/*z*): calcd, 194.1176 [M + H]^+^; found, 194.1182 [M + H]^+^.

### Synthesis of 4′-Methyl-[2,2′-bipyridine]-4-carbaldehyde (**4**)

Compound **4** was synthesized by following the general procedure as reported before.^24^ A mixture of 4,4′-dimethyl-2,2′-bipyridine (3 g, 0.016 mol) and selenium dioxide (1.98 g, 0.0179 mol) was suspended in degassed 1,4-dioxane solution (100 mL). The reaction mixture was heated to reflux for 40 h under a nitrogen atmosphere, resulting in a yellow solution with black metallic selenium precipitated. The resulting reaction was filtered hot and the filtrate was concentrated by rotary evaporator. The residue was dissolved again in a hot ethyl acetate solution for 30 min, and filtered in hot to remove any solid impurities. The filtrate was then extracted with 0.1 M Na_2_CO_3_ (2 × 100 mL) to remove carboxylic acid, and then with 0.3 M Na_2_S_2_O_5_ (3 × 100 mL) to form an aldehyde bisulfite. Combined the aqueous bisulfite fraction and adjusted the pH to around 10 by adding 1 M Na_2_CO_3_ to release aldehyde, then extracted with dichloromethane (4 × 100 mL). The organic fraction was collected and the solvent was removed by rotary evaporator to afford compound **4** as a white solid. Yield: 24%. ^1^H NMR (400 MHz, CDCl_3_, 298 K): δ = 10.19 (s, 1H), 8.90 (d, *J* = 4.9 Hz,1H), 8.84 (s, 1H), 8.58 (d, *J* = 4.9 Hz, 1H), 8.29 (s, 1H), 7.73 (s, *J* = 4.6 Hz, 1H), 7.21 (d, *J* = 4.6 Hz, 1H), 2.47 (s, 3H) ppm. ESI-TOF Mass (*m*/*z*) in methanol (*m*/*z*): calcd, 199.0866 [M + H]^+^; found, 199.0858 [M + H]^+^.

### Synthesis of 5,5-Difluoro-1,3,7,9-tetramethyl-10-(4′-methyl-[2,2′-bipyridin]-4-yl)-5*H*-4l4,5l4-dipyrrolo­[1,2-*c*:2′,1′-*f*]­[1,3,2]­diazaborinine (**5**)

The compound 5 was synthesis according to the literature procedure from Li Quan et al.^25^ A mixture of 4 (500 mg, 2.52 mmol) and 2,4-dimethylpyrrole (0.57 mL, 5.55 mmol) in anhydrous dichloromethane (50 mL) was combined with trifluoracetic acid (96 μL, 1.26 mmol) under nitrogen atmosphere, and the reaction was stirred for 4 h at room temperature. A suspension of 2,3-dichloro-5,6-dicyano-1,4-benzoquinone (DDQ, 572.04 mg, 2.52 mmol) in the solution of CH_2_Cl_2_ (3 mL) and tetrahydrofuran (3 mL) was then added into the reaction mixture and stirred for another 3 h at room temperature. After addition of Et_3_N (13.6 mL), the reaction was stirred for another 30 min. Last, BF3·OEt_2_ (13.6 mL) was added dropwise into the mixture under ice-cold condition and stirred for 30 min before warming up to room temperature, and the reaction was stirred overnight at room temperature. The resulting reaction was dissolved in CH_2_Cl_2_ and extracted with saturated Na_2_CO_3_ (3 × 100 mL) solution in order to eliminate the hydroquinone byproduct, and washed with brine (3 × 100 mL). The organic portion was combined and dried over anhydrous MgSO_4_, then evaporated under vacuum to get a crude product. The residue was purified by column chromatography with EtOAc/Hex (2:1) to yield 5 as a red solid. Yield: 34%. ^1^H NMR (400 MHz, CDCl_3_, 298 K): δ = 8.82 (d, *J* = 4.9 Hz, 1H); 8.48 (m, 2H); 8.30 (s, 1H); 7.30 (dd, *J* = 1.6, 4.9 Hz, 1H); 7.16 (d, *J* = 4.8 Hz, 1H); 5.99 (s, 2H); 2.56 (s, 6H); 2.45 (s, 3H); 1.46 (s, 6H) ppm. ^13^C NMR (125 MHz, CDCl_3_, 298 K): δ = 157.3, 156.3, 154.8, 149.8, 149.2, 148.3, 144.5, 142.7, 138.2, 130.4, 125.2, 123.0, 121.9, 121.6, 120.9, 43.3, 21.2, 14.9, 14.6, 11.0 ppm. ESI-TOF Mass (*m*/*z*) in methanol (*m*/*z*): calcd, 417.2057 [M + H]^+^; found, 417.2062 [M + H]^+^.

### Synthesis of (*E*)-4-(2-(5,5-Difluoro-1,7,9-trimethyl-10-(4′-methyl-[2,2′-bipyridin]-4-yl)-5*H*-4l4,5l4-dipyrrolo­[1,2-*c*:2′,1′-*f*]­[1,3,2]­diazaborinin-3-yl)­vinyl)-*N*-(2-Ethoxyethyl)­aniline (**B3**)

B3 was prepared by a Knoevenagel type condensation of compound 5 and compound 3. An anhydrous toluene solution (45 mL) combined with compound 5 (300 mg, 0.72 mmol), compound 3 (152.94 mg, 0.792 mmol), piperidine (1.377 mL, 13.98 mmol) and anhydrous 4-methylbenzenesulfonic acid (TsOH, 0.744 mL, 5.364 mmol) was refluxed at 145 °C for 12 h in a Dean–Stark apparatus. The solvent was removed by rotary evaporator to afford crude product. The residue was separated by column chromatography with EtOAc/Hex (2:1) to afford B3 as a dark blue solid. Yield: 39%. ^1^H NMR (400 MHz, CDCl_3_, 298 K): δ = 8.82 (d, *J* = 4.5 Hz, 1H); 8.52 (d, *J* = 5 Hz, 1H); 8.50 (s, 1H); 8.31 (s, 1H); 7.47 (m, 3H); 7.34 (dd, *J* = 1.7, 4.8 Hz, 1H); 7.21 (d, *J* = 16 Hz, 1H); 7.16 (d, *J* = 5.6 Hz, 1H); 6.61 (m, 3H); 5.98 (s, 1H); 3.66 (t, *J* = 5.1 Hz, 2H); 3.55 (q, *J* = 7.2 Hz, 2H); 3.35 (t, *J* = 5.3 Hz, 2H); 2.59 (s, 3H); 2.47 (s, 3H); 1.47 (s, 3H); 1.24 (m, 3H) ppm. ^13^C NMR (125 MHz, CDCl_3_, 298 K): δ = 157.1, 155.3, 154.9, 153.6, 149.6, 149.4, 149.2, 148.2, 144.8, 142.3, 140.3, 138.3, 135.3, 129.5, 125.8, 125.1, 123.5, 121.8, 121.3, 120.8, 114.3, 112.8, 68.6, 66.5, 43.2, 29.6, 21.2, 15.2, 15.1, 14.7, 14.6 ppm. ESI-TOF Mass (*m*/*z*) in methanol (*m*/*z*): calcd, 592.3054 [M + H]^+^; found, 592.3041 [M + H]^+^.

### Synthesis of *cis*-[Ru­(dmb)_2_Cl_2_]

A hot ethylene glycol solution (ethylene glycol/H_2_O = 3/1, 4 mL) that contained lithium chloride (20.47 mmol, 868 mg) and RuCl_3_·3H_2_O (3.82 mmol, 1 g) was heated to 110 °C under nitrogen atmosphere. After 15 min, dimethylbipyridine (7.7 mmol, 1.4 g) was added into the solution. Glucose (7.64 mmol, 1.376 mg) was added 15 min later, then after further 15 min, added l-ascorbic acid (2.04 mmol, 360.6 mg), which played as reducing agents to reduce Ru^3+^ to Ru^2+^. After refluxing for 30 min, brine (2 × 20 mL) was added and the precipitate was collected by filtration and washed with brine and a mixed solvent (toluene/ether/acetone = 14/5/1, 3 × 20 mL). Afterward, the black solid was sonicated in dichloromethane and filtered. This procedure was repeated for several times, then the purple solution was evaporated to afford Ru­(dmb)_2_Cl_2_. Yield: 61%. ^1^H NMR (400 MHz, DMSO-*d*_6_, 298 K): δ = 9.74 (d, *J* = 4.4 Hz, 2H); 8.45 (s, 2H); 8.31 (s, 2H); 7.56 (d, *J* = 4.4 Hz, 2H); 7.26 (s, 2H); 6.92 (d, *J* = 5.2 Hz, 2H); 2.60 (s, 6H); 2.37 (s, 6H) ppm. ESI-TOF Mass (*m*/*z*) in methanol (*m*/*z*): calcd, 540.0416 [M + H]^+^; found, 540.0419 [M + H]^+^.

### Synthesis of [Ru­(dmb)_2_(B3)]­(PF_6_)_2_ (**RuB3**)

Ru­(dmb)_2_Cl_2_ (49.3 mg, 0.091 mmol) and **B3** (50 mg, 0.084 mmol) were dissolved in a mixture of DCM, EtOH, H_2_O (v/v/v, 3/10/1) solution, which was stirred at 80 °C under nitrogen while shielded from light overnight. The reaction was monitored by Aluminum oxide TLC plate until a new green point appeared and remained steady. The solvent was then evaporated in vacuum and the remaining residue was purified by alumina column chromatography with a gradient elution (MeOH/DCM = 1:50 to 5:50). The resulting green band was collected, and the solvent was evaporated to obtain a crude product. The collected crude product was dissolved in the minimum MeOH, then slowly added saturated KPF_6_ aqueous solution, which resulting in the formation of a dark-green precipitate. The precipitate was purified by recrystallization from acetonitrile/ether to give the dark green solid. Yield: 61%. ^1^H NMR (400 MHz, CD_3_CN, 298 K): δ = 8.54 (s, 1H); 8.40–8.31 (m, 5H); 7.90–7.83 (m, 1H); 7.72–7.18 (m, 18H); 6.80 (d, *J* = 50 Hz, 1H); 6.71–6.66 (m, 1H); 3.59 (t, *J* = 5.2 Hz, 2H); 3.51 (q, *J* = 7 Hz, 2H); 3.31 (t, *J* = 5.2 Hz, 2H); 2.56–2.48 (m, 18H); 1.73 (d, *J* = 18.7 Hz, 3H); 1.27 (s, 3H); 1.17 (t, *J* = 3.5 Hz, 3H) ppm. ESI-TOF Mass (*m*/*z*) in methanol (*m*/*z*): calcd, 530.7019 [M+2PF6]^2+^; found, 530.7019 [M+2PF_6_]^2+^. Anal. Calcd for C_64_H_58_BF_14_N_9_O_2_P_2_Ru: C, 53.94; H, 4.10; N, 8.85%. Found: C, 52.61; H, 4.12; N, 8.94%.

### Cyclic Voltammetry

All electrochemical experiments data were collected on an electrochemical analyzer from CH Instruments (model 660D) at scan rate of 100 mV/s with a standard 3-electrode system: a silver wire reference electrode, a glassy carbon working electrode, and a platinum wire auxiliary electrode. Oxidation and reduction were performed by using 0.1 M tetrabutylammonium perchlorate (TBAP) and 1 mM substrate in dichloromethane. The supporting electrolyte was dried at 60 °C under vacuum for overnight and was stored inside a glovebox. In all cases, ferrocene was used as an internal standard, and all reduction potentials are reported with respect to the *E*_1/2_ of the Fc/Fc^+^ redox couple.

### Computational Method

All the density functional theory (DFT) calculations including full geometry optimization was performed using Gaussian 16 ^26^ program suites. The hybrid density functional B3LYP^27–29^ basis set and 6-31G­(d,p) was used for C, H, O, N, B and F atoms, coupled with LanL2DZ^30–32^ basis set that was used for Ru atom. Optimization of the molecular structure to tight convergence was completed at the B3LYP/LanL2DZ model chemistry. The time-dependent density functional theory (TD-DFT) was performed by using the polarizable continuum model for the methanol solvent.

## Results and Discussion

### Synthesis of the Ruthenium–Bipyridyl Complex

The synthesis of **RuB3** was meticulously achieved through a sequence of reactions, systematically depicted in Figure 1a. Initially, 4-vinylaniline was reacted with 1-bromo-2-ethoxyethane in the presence of potassium carbonate at 80 °C for 12 h, yielding the protected amine intermediate (1) (Figure S1). Subsequent Boc protection of this intermediate was performed using Boc anhydride in dioxane at 85 °C for 14 h to obtain compound (2) (Figure S2). The −NHCH_2_CH_2_OCH_2_CH_3_ substituent was selected as a flexible aniline-derived pendant group to increase molecular polarity and solubility while preserving an electron-donating conjugated linkage to the styryl–BODIPY framework, thereby supporting red-shifted absorption and biological applicability. Following this, compound (2) underwent ozonolysis at −78 °C, and the resultant product was treated with trifluoroacetic acid to produce the aldehyde intermediate (3) (Figure S3). This intermediate was further transformed into the pyrrole derivative (5) through a two-step process involving oxidation with selenium dioxide and a condensation reaction with 2,4-dimethylpyrrole in the presence of boron trifluoride etherate and triethylamine at room temperature (Figures S4–S6). The next critical step involved the synthesis of the BODIPY-bipyridine ligand (B3) by reacting the pyrrole derivative (5) with complex (3) and piperidine in toluene at 145 °C. This reaction successfully yielded the B3 ligand (Figures S7 and S8), which was then coordinated with ruthenium by reacting with RuCl_3_ in a solvent mixture of dichloromethane, methanol, and water at 80 °C. This final step produced the **RuB3** complex as a dication with two PF_6_^®^ counterions, highlighting a successful integration of the BODIPY and bipyridine moieties with ruthenium through a series of well-defined synthetic transformations (Figures S9 and S10). These compounds were also characterized using mass spectrometry (Figures S11–S18). The final-step isolated yield of RuB3 from B3 was 61%. The overall yield over the long linear sequence of the multistep synthesis was 3.54%. The FT-IR spectrum of B3 (Figure S19) displays characteristic transmittance peaks that are consistent with the expected functional groups in the ligand structure.

### Characterization and Properties of **RuB3**

The UV–vis spectra of B3 and **RuB3** demonstrate substantial electronic modulation after ruthenium coordination. B3 exhibits a strong visible absorption at 605 nm, consistent with the TD-DFT calculated transition at 602 nm, which is assigned to a HOMO → LUMO excitation with predominantly ligand-centered character involving the BODIPY ligand and dmb unit. Upon coordination to Ru, the resulting **RuB3** complex shows additional intense absorption features in the 427–446 nm region together with a broadened overall spectral profile. TD-DFT analysis attributes these bands to MLCT transitions (H-4 → L+1 and H-4 → LUMO) (Table S2), corresponding to electron transfer from the metal center to dmb and from the metal center to the BODIPY fragment. These results indicate that the Ru center introduces new low-lying charge-transfer states while preserving the strong visible absorption of the BODIPY framework. The coexistence of ligand-centered and MLCT transitions broadens the photoresponse of **RuB3** and is expected to enhance its light-harvesting and photochemical performance.

Figure S20 illustrates the time-dependent absorption spectra of 1,3-diphenylisobenzofuran (DBPF) under the influence of **RuB3** and Rose Bengal, monitored upon irradiation with 525 nm visible light. This experiment was designed to evaluate the singlet oxygen generation capabilities of **RuB3** compared to Rose Bengal, a well-known photosensitizer. Figure S20a shows the absorption spectrum of DBPF in the presence of **RuB3**. Over the course of 60 s, a gradual change in absorbance is observed, particularly in the range of 350–450 nm, indicating the production of singlet oxygen as a result of the interaction between **RuB3** and the light source. The absorbance change centered near 410 nm decreases over time, consistent with DPBF bleaching by singlet oxygen generated under irradiation. Figure S20b displays data for Rose Bengal. In contrast to **RuB3**, the increase in absorption at similar wavelengths is more pronounced, indicating a higher rate of singlet oxygen generation. From the linear fit of – ln­(A/A0) versus time at 525 nm (Figure 1f), B3 gave a degradation rate constant of 0.00183 min^–1^, corresponding to a ΦΔ value of approximately 0.07, which is lower than that of **RuB3** (0.12), supporting the conclusion that ruthenium coordination enhances photosensitization (Table S3). This kinetic analysis further substantiates that Rose Bengal facilitates a more rapid singlet oxygen generation than **RuB3**, albeit both compounds are effective in producing singlet oxygen under visible light irradiation. Time-resolved fluorescence measurements further showed that **RuB3** exhibits a substantially shorter fluorescence decay than B3 under 450 nm excitation (B3:0.334 ns, 95% and 0.956 ns, 5%; **RuB3**: 0.090 ns, 98% and 0.512 ns, 2%), indicating pronounced modulation of the excited-state deactivation pathway upon ruthenium coordination (Figure S21). This trend is consistent with the higher singlet oxygen quantum yield of **RuB3** relative to B3. However, it should be noted that although these data support improved photochemical performance upon ruthenium coordination, the present study does not include a direct triplet-state lifetime measurement and therefore does not constitute a complete excited-state mechanistic analysis.

The electrochemical properties of B3 and **RuB3** were investigated using cyclic voltammetry (CV), as shown in Figure 1g. The CV profiles were recorded in acetonitrile solution with tetrabutylammonium hexafluorophosphate as the supporting electrolyte. B3 exhibited a quasi-reversible redox couple with oxidation and reduction peaks at approximately 1.2 V and −1.5 V vs Fc/Fc^+^, respectively. Upon coordination with ruthenium, the CV profile of **RuB3** displayed significant changes. The **RuB3** complex showed a more defined redox behavior with oxidation peaks at approximately 1.1 V and reduction peaks at −1.6 V vs Fc/Fc^+^ (Table S4). The increased current density and enhanced reversibility of the redox peaks indicate improved electrochemical stability and charge-transfer characteristics in **RuB3** compared to B3 alone.

To gain a deeper mechanistic understanding of the observed ROS generation, the electrochemical properties of **RuB3** were correlated with TD-DFT calculations Figures 1c,d, S22 and Table S2. CV revealed a oxidation potential (*E*_ox_) at +0.85 V and reduction potential (*E*_red_) at −1.2 V (vs Fc/Fc^+^), yielding an experimental electrochemical bandgap of 2.05 eV. This aligns strongly with the calculated DFT HOMO–LUMO gap, validating the computational model. TD-DFT analysis confirms that the lowest-energy excitations are dominated by HOMO → LUMO transitions (orbitals 149 → 150). To distinguish between Type I and Type II ROS generation, the thermodynamics of the excited states were evaluated. The computed energy of the lowest triplet state (T_1_) is 1.41 eV. This value is substantially higher than the energy required for the excitation of ground-state molecular oxygen to singlet oxygen (0.98 eV), indicating a strong thermodynamic driving force for a Type II energy transfer mechanism. In contrast, the triplet excited-state oxidation potential (*E*_ox_*), calculated via the Rehm–Weller equation (*E*_ox_* = *E*_ox_ – *E*_T1_), is −0.56 V vs Fc/Fc^+^. Because this potential is generally insufficient to efficiently drive the one-electron reduction of oxygen, the Type I pathway is thermodynamically constrained. Consequently, these structural-electronic insights demonstrate that RuB3 operates predominantly as a Type II photosensitizer, aligning with the observed singlet oxygen generation in our DPBF assays.

### Reactive Oxygen Species Imaging in Cells

Figure 2a presents the results of a fluorescence microscopy study designed to assess the generation of reactive oxygen species (ROS) in MDA-MB-231 cells following treatment with **RuB3** under light conditions. The cells were stained with DAPI to visualize nuclei and DHR123 to detect ROS. In the absence of **RuB3** (control), cells exhibited minimal fluorescence from DHR123 both in dark and light conditions, indicating low basal levels of ROS. The nuclei were clearly stained with DAPI, showing typical morphology without significant alterations under both conditions. Upon treatment with 100 nM **RuB3**, a noticeable increase in green fluorescence from DHR123 was observed, indicating enhanced ROS generation. This effect was more pronounced under light exposure, suggesting that **RuB3** activates photodynamically to produce significant levels of ROS in the presence of light. The comparative analysis between dark and light conditions reveals that **RuB3**-mediated ROS generation is significantly light-dependent. The enhanced photodynamic activity under light exposure not only enhance ROS production but also potentially leads to oxidative stress, affecting cellular health and morphology.

**Figure 2:**
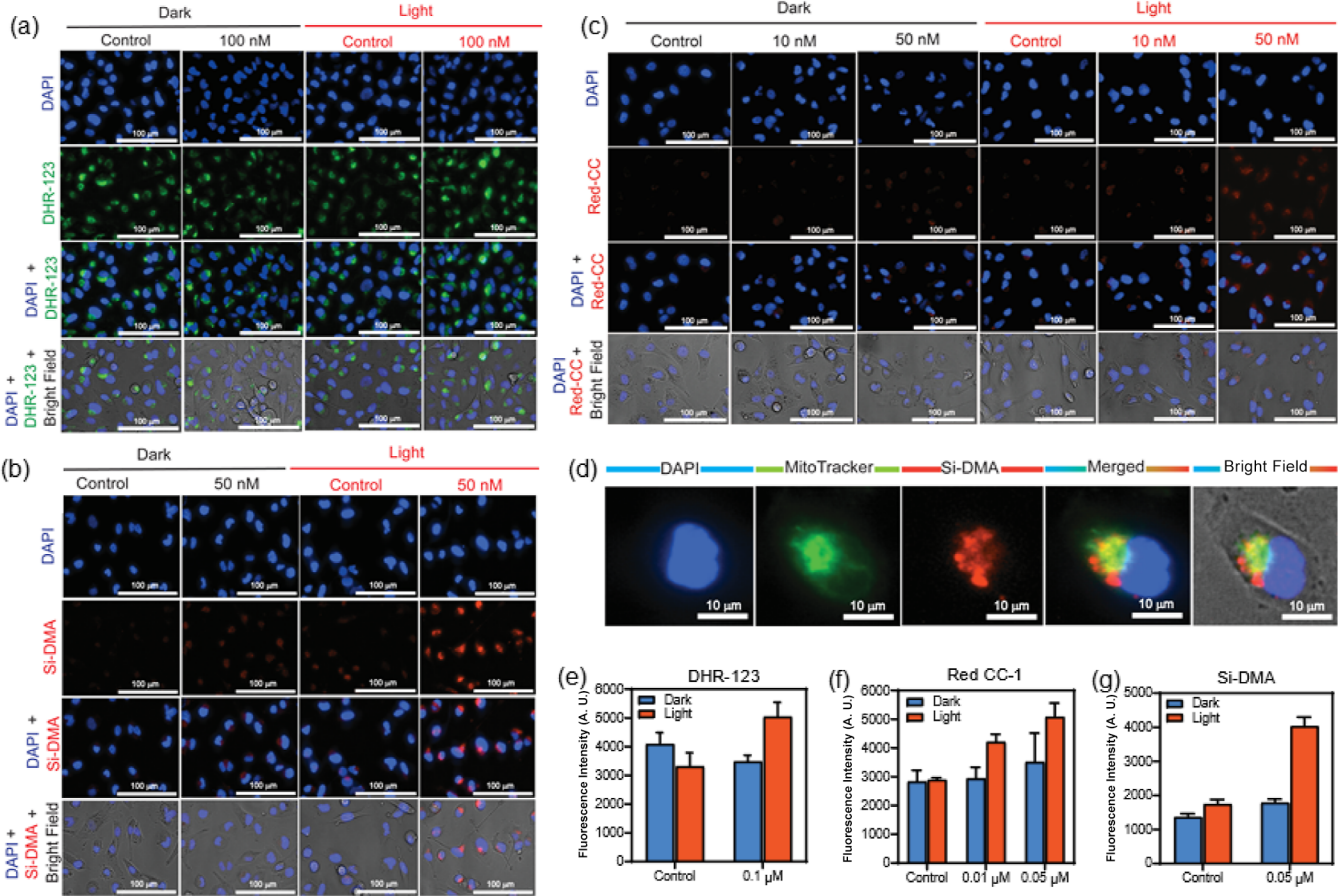
(a) Confocal fluorescence microscopy images showing intracellular ROS generation under different conditions. Cells were stained with DAPI (blue) for nuclei and DHR-123 (green) for ROS detection. Control and treated cells (100 nM) were analyzed under dark and light conditions. (b) Fluorescence images of cells stained with Si-DMA (red) for singlet oxygen detection, with DAPI (blue) marking nuclei. The experiment compares control and treated cells (50 nM) under dark and light conditions. (c) Fluorescence microscopy images of cells stained with Red-CC (red) to visualize singlet oxygen formation under different light conditions and treatment concentrations (10 nM and 50 nM). (d) Colocalization study showing the distribution of Si-DMA (red) in mitochondria (stained with MitoTracker, green) and nuclei (DAPI, blue), demonstrating singlet oxygen detected in mitochondrial. (e–g) Quantitative analysis of fluorescence intensities for different ROS detection probes under varying treatment conditions in dark and light settings. Data represent mean fluorescence intensity, with statistical comparisons highlighting the effect of light activation on ROS and singlet oxygen generation.

We also assessed the production of singlet oxygen using the Si-DMA probe. Among various reactive oxygen species, Si-DMA specifically detects singlet oxygen. In both dark and light conditions, control cells exhibited minimal Si-DMA fluorescence, indicating low baseline levels of singlet oxygen (Figure 2b). In the dark, cells treated with 50 nM of the compound showed a slight increase in Si-DMA fluorescence compared to control, suggesting a basal level of singlet oxygen generation even in the absence of light. However, this effect was dramatically amplified under light exposure, where treated cells exhibited robust Si-DMA fluorescence. This marked increase in red fluorescence under light conditions confirms the photosensitive nature of the compound, which significantly enhances singlet oxygen production when activated by light. To further probe the ROS profile of **RuB3**, additional experiments were performed using DHE and HPF to evaluate superoxide-associated and hydroxyl-radical-associated signals, respectively (Figure S23). Under dark conditions, both probes showed negligible fluorescence across the tested concentrations. In contrast, under light irradiation, **RuB3** induced clear and concentration-dependent increases in both DHE and HPF signals. These responses were detectable but less pronounced than the Si-DMA singlet oxygen signal, suggesting that **RuB3** predominantly undergoes Type II photosensitization while retaining a measurable Type I contribution under photoirradiation. To further probe the contribution of singlet oxygen, NaN_3_ was employed as a singlet oxygen scavenger (Figure S24). In the presence of NaN_3_, the photoinduced oxidative signal was markedly reduced, particularly the singlet-oxygen-associated fluorescence, supporting a major role of Type II photosensitization in the activity of **RuB3**.

We also examine the cytosolic redox potential using Red CC-1. RedoxSensor Red CC-1, which fluoresces upon oxidation in the presence of singlet oxygen, predominantly within mitochondria, was used to track the oxidative state of the cells (Figure 2c). The probe’s behavior under different conditions offers insights into the intracellular dynamics induced by **RuB3**. In the absence of **RuB3**, both dark and light conditions showed minimal RedoxSensor Red CC-1 fluorescence, indicating low levels of oxidative stress within the cells. A slight increase in RedoxSensor Red CC-1 fluorescence was observed as the concentration of **RuB3** increased, suggesting a basal level of ROS generation by **RuB3** even without light activation. Marked increases in fluorescence were evident with both 10 nM and 50 nM treatments under light exposure, indicating mitochondrial stress triggered by the photodynamic action of **RuB3**.

The results demonstrate that **RuB3** can induce oxidative stress within mitochondria, particularly under light exposure, which is consistent with the intended design of **RuB3** as a photodynamic therapy agent. The increase in RedoxSensor Red CC-1 fluorescence in mitochondria upon light activation indicates effective singlet oxygen generation by **RuB3**, aligning with its proposed mechanism of inducing cell death through mitochondrial dysfunction. This mitochondrial targeting is advantageous for cancer treatment strategies, where selective induction of apoptosis in cancer cells via mitochondrial disruption can significantly enhance treatment efficacy while potentially reducing side effects on normal tissues.

Colocalization analysis of the MitoTracker Green and Si-DMA channels gave a Pearson’s correlation coefficient of 0.896, indicating strong spatial overlap and supporting that photoinduced singlet oxygen generation occurs in close proximity to mitochondria (Figure S25). This localization is significant as mitochondria are key sites for cellular energy metabolism and apoptosis regulation. The ICP–MS results showed that the presence of **RuB3** in these organelles could imply potential mechanisms through which **RuB3** might exert its effects, possibly through the modulation of mitochondrial functions or induction of oxidative stress within these critical organelles (Table S5). ICP–MS analysis also revealed higher total intracellular ruthenium uptake in MDA-MB-231 cells than in H184B5F5/M10 cells after incubation with **RuB3** under identical conditions, suggesting that differential cellular accumulation may contribute to the stronger phototoxic response observed in the cancer cells. To further evaluate the solution stability of **RuB3**, the time-dependent UV–vis absorption spectrum was monitored over 24 h. **RuB3** exhibited good absorption stability, with only a slight decrease in absorbance at λ_max from 0.412 to 0.361 over 24 h, corresponding to 87.6% retention of the initial absorbance (Table S6).

### Cell Viability Under Dark and Light Conditions

Figures S26–S28 and Table S7 display the cell viability assays for **RuB3** across three different cancer cell lines: MDA-MB-231, MCF-7, and H184B5F5/M10, under both dark and light conditions. In the triple-negative breast cancer cell line MDA-MB-231, **RuB3** exhibited a significant reduction in cell viability under light irradiation. Under dark conditions, the compound showed minimal cytotoxicity, underscoring the light-activated nature of its therapeutic action. The sharp decline in viability in light conditions indicates **RuB3**’s potent phototoxic effects specific to cancer cells (Figure S29). Similar effects were observed in the MCF-7 cell line, a model for estrogen receptor-positive breast cancer. Interestingly, the light-exposed H184B5F5/M10 cells, a nontumorigenic mammary cell line, retained relatively high viability until higher concentrations, indicating that **RuB3**’s phototoxicity is less pronounced in noncancerous cells.

In MDA-MB-231, it exhibited an IC_50_ of 1.88 μM in dark conditions, significantly reduced to 0.50 μM under light exposure (Table S7). Similar to MDA-MB-231, the IC_50_ of MCF-7 decreased from 1.40 μM in the dark to 0.47 μM with light, highlighting effective light-induced activation against estrogen receptor-positive breast cancer cells. In contrast, H184B5F5/M10 showed the least difference in IC_50_ values between dark (1.94 μM) and light (1.64 μM) conditions, suggesting a moderate photodynamic effect in this nontumorigenic mammary cell line.

To further quantify the light-dependent cytotoxicity of **RuB3**, the photocytotoxicity index (PI = IC50, dark/IC50, light) was calculated from the IC50 values in Table S8. The resulting PI values were 3.76 for MDA-MB-231, 2.98 for MCF-7, and 1.18 for H184B5F5/M10, indicating stronger photoinduced cytotoxic enhancement in cancer cells than in the nontumorigenic cell line. As a control, Ru­(bpy)_3_Cl_2_ was also evaluated under the same dark and light conditions (Table S7). Ru­(bpy)_3_Cl_2_ showed negligible cytotoxicity in all tested cell lines, with IC_50_ values greater than 80 μM under both dark and light irradiation. This result indicates that the Ru­(bpy)_3_Cl_2_ unit alone does not contribute significant cytotoxicity or photocytotoxicity under the present experimental conditions. This is mainly attributed to the low cellular uptake, which has been previously reported by several groups.^33^ Compared with previously reported Ru-based anticancer compounds and cisplatin, **RuB3** exhibited markedly lower light IC_50_ values in both MDA-MB-231 and MCF-7 cells, indicating stronger photocytotoxic potency under irradiation (Table S9).

To further assess the biological relevance of **RuB3** under oxygen-limited conditions, additional experiments were performed under hypoxia (Figure S30). Compared with normoxia, hypoxic conditions led to lower but still clearly detectable intracellular ROS generation and cytotoxicity upon photoirradiation. These results indicate that **RuB3** retains partial photodynamic activity under oxygen-limited conditions, although with reduced efficacy relative to normoxia. This behavior is consistent with the observed mixed Type I/Type II ROS profile, in which a measurable Type I contribution may help preserve activity when oxygen availability is reduced.

### Flow Cytometry Analysis of Apoptosis Induction by **RuB3**

The apoptotic and necrotic effects of **RuB3** in MDA-MB-231 cells were analyzed using Annexin V-FITC/PI staining, followed by flow cytometry. Figure 3a presents the flow cytometry results for cells treated with **RuB3** at different concentrations (10 nM and 50 nM) in the presence and absence of light (525 nm) irradiation.

**Figure 3:**
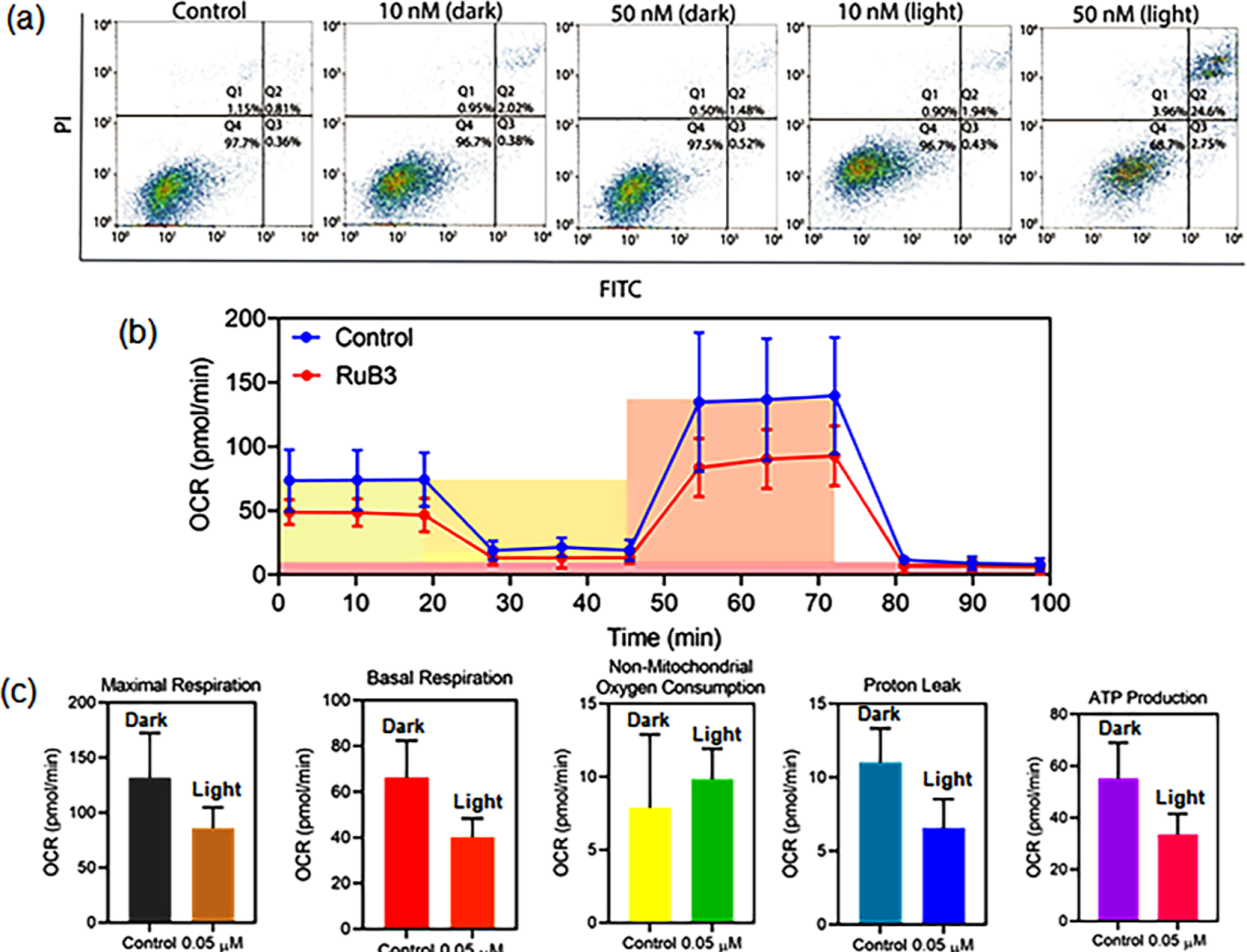
(a) Flow cytometry analysis of cell viability after **RuB3** treatment, showing PI (propidium iodide) and FITC staining to assess apoptotic and necrotic cell populations. (b) Oxygen consumption rate (OCR) measurements comparing control and **RuB3**-treated cells over time, with distinct phases highlighted for basal respiration (yellow) and maximal respiration (red) for control and **RuB3**-treated cells at 50 nM. (c) Quantitative analysis of mitochondrial respiration parameters, including basal respiration, ATP production, proton leak, and maximal respiration, demonstrating the impact of **RuB3** treatment.

In the control group and cells treated with 10 nM **RuB3** without light irradiation, the majority of cells remained viable, as indicated by low Annexin V and PI staining. However, in the 10 nM **RuB3**-treated cells with PDT, there was a significant increase in Annexin V-positive/PI-negative cells, indicating early apoptosis. The percentage of early apoptotic cells increased from 2.3% in the control to 29.4% in the PDT-treated group.

For cells treated with 50 nM **RuB3**, a notable increase in both early apoptotic (Annexin V-positive/PI-negative) and late apoptotic/necrotic (Annexin V-positive/PI-positive) cells was observed under light irradiation. The percentage of early apoptotic cells increased from 2.3% in the control to 44.6% in the PDT-treated group, and late apoptotic/necrotic cells increased from 1.0 to 19.0%.

These results indicate that **RuB3** effectively induces apoptosis in MDA-MB-231 cells upon light activation, with a dose-dependent increase in apoptotic cell populations. The flow cytometry data support the potential of **RuB3** as an effective photosensitizer for inducing cell death through apoptosis, further validating its use in photodynamic therapy.

To further validate apoptotic signaling, Western blot analysis was performed for Bcl-2, Bax, cleaved caspase-9, cleaved caspase-3, and cleaved PARP. Relative to the untreated control and dark-treated groups, the light-treated RuB3 groups showed reduced Bcl-2 and increased Bax, cleaved caspase-9, cleaved caspase-3, and cleaved PARP, supporting activation of a mitochondria-associated apoptotic pathway under PDT conditions (Figure S31).

The effect of **RuB3** on mitochondrial respiration was assessed using the Mito Stress Test in MDA-MB-231 cells. Figure 3b shows the oxygen consumption rate (OCR) profile over time, while Figure 3c present the key parameters for mitochondrial respiration, including basal respiration, nonmitochondrial oxygen consumption, proton leak, ATP production, and maximal respiration.

The OCR profile in Figure 3c indicates a significant decrease in mitochondrial respiration upon treatment with **RuB3**. The addition of oligomycin (Oligo), FCCP, and rotenone/antimycin A (RO/AA) revealed distinct phases of mitochondrial activity, highlighting the impact of **RuB3** on mitochondrial function. Basal respiration and ATP production were significantly reduced in **RuB3**-treated cells compared to the control, indicating impaired mitochondrial function. Nonmitochondrial oxygen consumption and proton leak also showed a reduction, suggesting decreased overall cellular respiration. Maximal respiration was notably lower in **RuB3**-treated cells, further confirming the inhibitory effects on mitochondrial function.

These results demonstrate that **RuB3** significantly impairs mitochondrial respiration, leading to decreased ATP production and overall cellular energy metabolism. The disruption of mitochondrial function likely contributes to the observed apoptotic effects in **RuB3**-treated cells upon light activation.

### Live Cell Imaging and Morphological Changes

Figure 4 presents a time-lapse series captured using the Nanolive imaging system, which illustrates the dynamic cellular responses and morphological changes in MDA-MB-231 cells following treatment with **RuB3**. Utilizing the high-content imaging capabilities of the Nanolive system, which provides detailed real-time visualization of cellular processes without the need for invasive dyes or labels, we tracked the effects of **RuB3** on cancer cell morphology over a period of approximately 800 min (Video S1).

**Figure 4:**
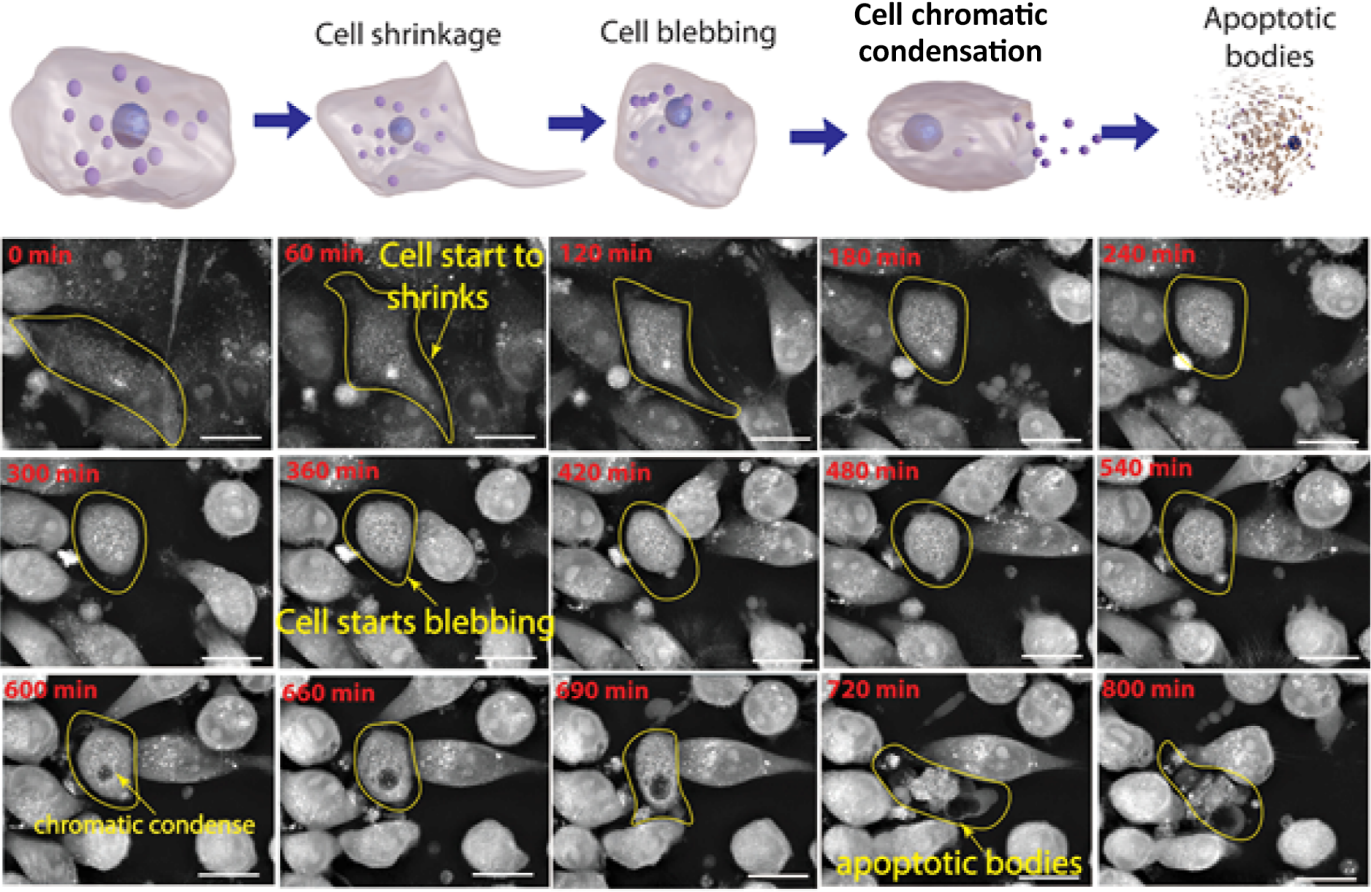
Time-lapse fluorescence microscopy images showing morphological changes in cells undergoing apoptosis upon **RuB3** treatment, with outlined cells indicating progressive cell shrinkage, blebbing, and chromatin condensation over 800 min.

For the initial response (0 to 120 min), the initial images show MDA-MB-231 cells with typical morphology. Following the introduction of **RuB3**, no immediate morphological changes were observed, suggesting that the initial interaction of **RuB3** with the cells is subtle or occurs at a molecular level not immediately visible via morphology alone.

In the intermediate stages (180 to 240 min), by 180 min, slight changes in cell morphology begin to appear, including cell rounding and reduced adherence, which are indicative of the onset of cellular stress or early apoptotic events.

In the advanced effects (300 to 800 min), as the incubation time increases, more pronounced morphological changes are evident. Cells show significant rounding, loss of adherence, and the appearance of apoptotic bodies by 800 min. These changes become more pronounced by 720 min, with nearly all cells displaying signs of advanced apoptosis.

The time-dependent morphological changes observed suggest that **RuB3** induces apoptosis in MDA-MB-231 cells. This is likely mediated through the generation of reactive oxygen species (ROS), which lead to mitochondrial dysfunction and subsequent apoptotic cascades. The ability of **RuB3** to induce significant morphological changes, culminating in cell death, underscores its potential as a therapeutic agent in cancer treatment, particularly for aggressive types such as triple-negative breast cancer. The live cell imaging results provides invaluable insights into the timeline and progression of **RuB3**’s effects at the cellular level.

## Conclusions

This study has elucidated the significant potential of **RuB3** as a photodynamic therapy agent in targeting cancer cells, particularly the MDA-MB-231 cell line. Through a series of meticulous experiments involving live cell imaging, fluorescence microscopy, and flow cytometry, we have demonstrated that **RuB3** induces apoptosis and morphological changes in cancer cells, with these effects being markedly enhanced under light exposure. The localization studies using DAPI, MitoTracker, and LysoTracker highlighted **RuB3**’s ability to target and accumulate within mitochondria and lysosomes, initiating cellular stress and apoptosis through the generation of reactive oxygen species.

The Annexin V-FITC/PI staining results provided quantitative evidence of the increased apoptotic and necrotic cell populations following **RuB3** treatment under photodynamic conditions, suggesting that **RuB3**’s activity is both concentration and light-dependent. Additionally, the real-time visualization of cellular interactions with **RuB3** offered significant insights into the dynamic cellular processes over time, confirming the compound’s capability to induce significant morphological transformations leading to cell death.

The implications of these findings are profound, underscoring **RuB3**’s potential as an effective therapeutic tool in the fight against cancer, particularly types that are resistant to conventional therapies. However, the selective activation of **RuB3** under light conditions also highlights its potential for minimizing side effects in normal tissues, a significant advantage in reducing the overall toxicity of cancer treatments.

## Supplementary Material





## Notes

Experimental details for singlet oxygen quantum yield analysis, cell viability assays, intracellular ROS detection, apoptosis assays, subcellular localization studies, intracellular superoxide and hydroxyl-radical-associated ROS detection, mitochondrial respiration measurements, live-cell imaging, Western blot analysis, and photocytotoxicity assays under normoxic and hypoxic conditions are provided. Also includes characterization data, including NMR, high-resolution mass, ATR-IR, photophysical, electrochemical, and computational data, as well as singlet oxygen and ROS detection results, cell viability, apoptosis, subcellular localization, Western blot analysis, hypoxia/normoxia fluorescence imaging, and additional supporting tables (PDF)
The effects of **RuB3** on cancer cell morphology over a period of approximately 800 min (MP4)

## References

[1] Pass H. I. (1993). Photodynamic
Therapy in Oncology: Mechanisms and Clinical Use. J. Natl. Cancer Inst..

[2] Baptista M. S., Cadet J., Di Mascio P., Ghogare A. A., Greer A., Hamblin M. R., Lorente C., Nunez S. C., Ribeiro M. S., Thomas A. H., Vignoni M., Yoshimura T. M. (2017). Type I
and Type II Photosensitized Oxidation Reactions: Guidelines and Mechanistic
Pathways. Photochem. Photobiol..

[3] Medina M. A., Oza G., Sharma A., Arriaga L. G., Hernández Hernández J. M., Rotello V. M., Ramirez J. T. (2020). Triple-Negative Breast Cancer: A
Review of Conventional and Advanced Therapeutic Strategies. Int. J. Environ. Res. Public Health.

[4] Jiang Q., Zhang M., Sun Q., Yin D., Xuan Z., Yang Y. (2021). Enhancing the Antitumor Effect of Doxorubicin with Photosensitive
Metal–Organic Framework Nanoparticles against Breast Cancer. Mol. Pharmaceutics.

[5] Wang J., Wu H., Zhao Q., Zou Y., Ding D., Yin H., Xu H. (2022). Aggregation-Induced
Emission Photosensitizer Synergizes Photodynamic
Therapy and the Inhibition of the NF-κB Signaling Pathway to
Overcome Hypoxia in Breast Cancer. ACS Appl.
Mater. Interfaces.

[6] Djeungoue-Petga M.-A., Lurette O., Jean S., Hamel-Côté G., Martín-Jiménez R., Bou M., Cannich A., Roy P., Hebert-Chatelain E. (2019). Intramitochondrial Src kinase links
mitochondrial dysfunctions and aggressiveness of breast cancer cells. Cell Death Dis..

[7] Tian M., Chen W., Wu Y., An J., Hong G., Chen M., Song F., Zheng W.-h., Peng X. (2022). Liposome-Based
Nanoencapsulation of a Mitochondria-Stapling Photosensitizer for Efficient
Photodynamic Therapy. ACS Appl. Mater. Interfaces.

[8] Feng G., Liu J., Zhang C.-J., Liu B. (2018). Artemisinin and AIEgen Conjugate
for Mitochondria-Targeted and Image-Guided Chemo- and Photodynamic
Cancer Cell Ablation. ACS Appl. Mater. Interfaces.

[9] Yuan P., Deng F.-A., Liu Y.-B., Zheng R.-R., Rao X.-N., Qiu X.-Z., Zhang D.-W., Yu X.-Y., Cheng H., Li S.-Y. (2021). Mitochondria Targeted
O_2_ Economizer to Alleviate Tumor
Hypoxia for Enhanced Photodynamic Therapy. Adv.
Healthc. Mater..

[10] Chakrabortty S., Agrawalla B. K., Stumper A., Vegi N. M., Fischer S., Reichardt C., Kögler M., Dietzek B., Feuring-Buske M., Buske C., Rau S., Weil T. (2017). Mitochondria Targeted
Protein-Ruthenium Photosensitizer for Efficient Photodynamic Applications. J. Am. Chem. Soc..

[11] Zeng L., Gupta P., Chen Y., Wang E., Ji L., Chao H., Chen Z.-S. (2017). The Development of Anticancer Ruthenium­(ii)
Complexes: from Single Molecule Compounds to Nanomaterials. Chem. Soc. Rev..

[12] van
Rixel V. H. S., Siewert B., Hopkins S. L., Askes S. H. C., Busemann A., Siegler M. A., Bonnet S. (2016). Green Light-Induced
Apoptosis in Cancer Cells by A Tetrapyridyl Ruthenium Prodrug Offering
Two Trans Coordination Sites. Chem. Sci..

[13] Ni Y., Wu J. (2014). Far-Red and Near Infrared
BODIPY Dyes: Synthesis and Applications
for Fluorescent pH Probes and Bio-imaging. Org.
Biomol. Chem..

[14] Loudet A., Burgess K. (2007). BODIPY Dyes and Their
Derivatives: Syntheses and Spectroscopic
Properties. Chem. Rev..

[15] Li H., Hao Y.-H., Feng W., Song Q.-H. (2020). Rapid and Sensitive
Detection of Nitric Oxide by a BODIPY-Based Fluorescent Probe in Live
Cells: Glutathione Effects. J. Mater. Chem.
B.

[16] Aksakal N. E., Eçik E. T., Kazan H. H., Çiftçi G. Y., Yuksel F. (2019). Novel Ruthenium­(II)
and Iridium­(III) BODIPY Dyes: Insights
into Their Application in Photodynamic Therapy In Vitro. Photochem. Photobiol. Sci..

[17] Kursunlu A. N. (2014). Porphyrin–BODIPY
Combination: Synthesis, Characterization, and Antenna Effect. RSC Adv..

[18] Paul S., Kundu P., Kondaiah P., Chakravarty A. R. (2021). BODIPY-Ruthenium­(II)
Bis-Terpyridine Complexes for Cellular Imaging and Type-I/-II Photodynamic
Therapy. Inorg. Chem..

[19] Zhou Q.-X., Lei W.-H., Hou Y.-J., Chen Y.-J., Li C., Zhang B.-W., Wang X.-S. (2013). BODIPY-Modified
Ru­(II) Arene Complex—A
New Ligand Dissociation Mechanism and a Novel Strategy to Red-Shift
the Photoactivation Wavelength of Anticancer Metallodrugs. Dalton Trans..

[20] Yu X., Gao F., Zhao W., Lai H., Wei L., Yang C., Wu W. (2022). BODIPY-Conjugated Bis-Terpyridine Ru­(II) Complexes Showing Ultra-Long
Luminescence Lifetimes and Applications to Triplet–Triplet
Annihilation Upconversion. Dalton Trans..

[21] Paul S., Sahoo S., Sahoo S., Jayabaskaran C., Chakravarty A. R. (2022). Bichromophoric BODIPY and Biotin
Tagged Terpyridyl
Ruthenium­(II) Complexes for Cellular Imaging and Photodynamic Therapy. Eur. J. Inorg. Chem..

[22] Aksoy B. T., Özcan E., Bulut O., Kazan H. H., Çoşut B. (2025). Synthesis,
Photophysical Properties, and Photodynamic Therapy Efficacies of Meso-Pyridine
BODIPYs and Their Ruthenium Complexes. Appl.
Organomet. Chem..

[23] Paul S., Bera A. (2026). Enriching BODIPY Triplet States via Ruthenium­(II) Conjugation for
Improved Photodynamic Therapy. J. Med. Chem..

[24] Lebedeva M. A., Chamberlain T. W., Scattergood P. A., Delor M., Sazanovich I. V., Davies E. S., Suyetin M., Besley E., Schröder M., Weinstein J. A., Khlobystov A. N. (2016). Stabilising the Lowest-Energy Charge-Separated
State in a {Metal Chromophore–Fullerene} Assembly: A Tuneable
Panchromatic Absorbing Donor–Acceptor Triad. Chem. Sci..

[25] Quan L., Sun T., Lin W., Guan X., Zheng M., Xie Z., Jing X. (2014). BODIPY Fluorescent Chemosensor for Cu^2+^ Detection and
Its Applications in Living Cells: Fast Response and High Sensitivity. J. Fluoresc..

[26] Frisch, M. J. ; Trucks, G. W. ; Schlegel, H. B. ; Scuseria, G. E. ; Robb, M. A. ; Cheeseman, J. R. ; Scalmani, G. ; Barone, V. ; Petersson, G. A. ; Nakatsuji, H. ; Li, X. ; Caricato, M. ; Marenich, A. V. ; Bloino, J. ; Janesko, B. G. ; Gomperts, R. ; Mennucci, B. ; Hratchian, H. P. ; Ortiz, J. V. ; Izmaylov, A. F. ; Sonnenberg, J. L. ; Williams; Ding, F. ; Lipparini, F. ; Egidi, F. ; Goings, J. ; Peng, B. ; Petrone, A. ; Henderson, T. ; Ranasinghe, D. ; Zakrzewski, V. G. ; Gao, J. ; Rega, N. ; Zheng, G. ; Liang, W. ; Hada, M. ; Ehara, M. ; Toyota, K. ; Fukuda, R. ; Hasegawa, J. ; Ishida, M. ; Nakajima, T. ; Honda, Y. ; Kitao, O. ; Nakai, H. ; Vreven, T. ; Throssell, K. ; Montgomery, J. A., Jr ; Peralta, J. E. ; Ogliaro, F. ; Bearpark, M. J. ; Heyd, J. J. ; Brothers, E. N. ; Kudin, K. N. ; Staroverov, V. N. ; Keith, T. A. ; Kobayashi, R. ; Normand, J. ; Raghavachari, K. ; Rendell, A. P. ; Burant, J. C. ; Iyengar, S. S. ; Tomasi, J. ; Cossi, M. ; Millam, J. M. ; Klene, M. ; Adamo, C. ; Cammi, R. ; Ochterski, J. W. ; Martin, R. L. ; Morokuma, K. ; Farkas, O. ; Foresman, J. B. ; Fox, D. J. Gaussian 16, Rev. C.01; Gaussian Inc.: Wallingford, CT, 2016.

[27] Lee C., Yang W., Parr R. G. (1988). Development
of the Colle–Salvetti
Correlation-Energy Formula into a Functional of the Electron Density. Phys. Rev. B.

[28] Yang W., Parr R. G., Lee C. (1986). Various Functionals for the Kinetic
Energy Density of an Atom or Molecule. Phys.
Rev. A:At., Mol., Opt. Phys..

[29] Kohn W., Becke A. D., Parr R. G. (1996). Density Functional
Theory of Electronic
Structure. J. Phys. Chem. C.

[30] Hay P. J., Wadt W. R. (1985). Ab Initio Effective Core Potentials for Molecular Calculations.
Potentials for the Transition Metal Atoms Sc to Hg. J. Chem. Phys..

[31] Wadt W. R., Hay P. J. (1985). Ab initio effective core potentials for molecular calculations
Potentials for main group elements Na to Bi. J. Chem. Phys..

[32] Hay P. J., Wadt W. R. (1985). Ab Initio Effective Core Potentials for Molecular Calculations.
Potentials for the Transition Metal Atoms Sc to Hg. J. Chem. Phys..

[33] Durr, H. ; Bouas-Laurent, H. Photochromism: Molecules and Systems; Elsevier, 2003.

