# Supplementary material for: A Ruthenium–BODIPY Photosensitizer for Light-Triggered Apoptosis in Triple-Negative Breast Cancer Cells
Source: Inorg Chem. 2026 Jun 26;65(27):15597–607. doi: 10.1021/acs.inorgchem.6c01479 (PMC13370859; doi:10.1021/acs.inorgchem.6c01479)
Supplement: Supplementary file 1 [file ic6c01479_si_001.pdf]

# Supporting Information

## A Ruthenium–BODIPY Photosensitizer for Light-Triggered Apoptosis in Triple-Negative Breast Cancer Cells

Ting-Hsuan Wang,[‡a] Chia-Hsuan Lin,[‡b] Eng Zhi Sim,[b] Melody Cai-Syaun Wu,[b] Jack Hau-Ting Wei,[b] Wan Tien Huang,[b] Ricky Yu-Syun Fan,[c] Siao-Cian Pan,[c] Sangeeta,[d] Rakesh Ganguly,[d*] Kien Voon Kong[b,e*] and Tiow-Gan Ong[a*]

[a]School of Science and Engineering, The Chinese University of Hong Kong, Shenzhen, Guangdong, 518172 China

[b]Department of Chemistry, National Taiwan University, No. 1, Sec. 4, Roosevelt Rd., Taipei 10617, Taiwan (R.O.C.).

[c]Immune Research Core, Department of Medical Research, National Taiwan University Hospital, No.7, Chung Shan S. Rd., Zhongzheng Dist., Taipei City 100225, Taiwan (R.O.C.).

[d]Shiv Nadar Institute of Eminence, Delhi NCR, India

[e]Center For Emerging Material and Advanced Devices, National Taiwan University, Taipei10617, Taiwan

[‡]These authors contributed equally.

Corresponding author: rakesh.ganguly@snu.edu.in; kvkong@ntu.edu.tw; tgong@cuhk.edu.cn

**Experimental Section**

**Singlet Oxygen Quantum Yield Analysis**

RuB3(5μM) was mixed with the DPBF (90 μM) in a methanol solution. The mixture solution was stored in the dark and purged with oxygen for 15 min. The solution mixtures were then illuminated at the wavelength of 525 nm and monitored at 410 nm every 10 s. The slopes were calculated and compared to the reference Rose Bengal ($\Phi_\Delta$ = 0.76 in methanol).

**Cell Culture**

MDA-MB-231, MCF-7 and H184B5F5/M10 cells were grown in DMEM/ F-12 medium. The media were supplemented with 10% of Fetal Bovine Serum (FBS) and 1% of penicillin/streptomycin. All cells were grown at 37 °C in a humidified atmosphere with 5% CO2 humidified incubator and passaged regularly at approx. 90% confluence.

**Cell Viability Assay**

MDA-MB-231, MCF-7 and H184B5F5/M10 cells ($1\times10^4$ per well) were seeded in a flat-bottomed 96-well plate and cultured overnight. All media were then removed from wells and incubated with 100 μL

of RuB3 to triplicate wells with various concentrations. The plates were labelled for either no irradiation (dark) or radiation (light). The dark plates were incubated for 24 h, whereas the light plates were first incubated for 2 h, then exposed to the 525 nm light for 10 min, then continue incubated for 24 h. The media were discarded and the cells were washed twice with DPBS. After replacing the medium with serum-containing media, 1 μL NucBlue Live reagent (staining the nuclei of all cells) and 1 μL NucGreen Dead reagent (staining only the nuclei of dead cells with compromised plasma membranes) were added into each well. After 20 min of incubations, the cells were rinsed with the DPBS three times and live cell images were acquired on BioTek Cytation 5 Cell Imaging Multi-Mode Reader. Cell viability was calculated by; CV = [(total number of cells (DAPI fluorescence) - (Number of dead cells (GFP fluorescence)/ total number of cells (DAPI fluorescence)] *100%. Cell viability of wells was determined in triplicate and the data collected was analysed with GraphPad Prism 9.

**Intracellular Reactive Oxygen Species (ROS) Detection with RedoxSensor™ Red CC-1**

MDA-MB-231 cells ($1\times10^4$ per well) were seeded in 96-well plates for 24 h before live cell imaging experiments. All media were removed from wells and the cells were treated with RuB3 (0.01 μM and 0.05 μM) for 2 h. For light treatment, RuB3 was first incubated for 2 h, then added the RedoxSensor™ Red CC-1 probe (5 μM) for 15 minutes incubation, according to the manufacturer's instructions (Invitrogen), followed by the 525 nm light irradiation for 10 minutes. The cells were washed with DPBS three times and imaged in serum-free DMEM media. Control wells were treated with DMSO alone (dark/light). The fluorescence images of the ROS generation of RuB3 during PDT treatment on MDA-MB-231 cells were captured on BioTek Cytation 5 Cell Imaging Multi-Mode Reader and the image is analysed by using BioTek Gen5 Software. For all light-dependent biological evaluations, cells were irradiated using a Kessil lamp (427 nm, 10 Watt, distance = 10 cm, light intensity = 10 mW $cm^{-2}$, irradiated area = 7.0 $cm^2$). Cells were irradiated for 10 minutes, resulting in a total light dose (fluence) of 0.857 $J/cm^2$. Control cells in the dark groups were kept in the incubator under identical conditions without light exposure.

**Intracellular Reactive Oxygen Species (ROS) Detection with Dihydrorhodamine 123**

MDA-MB-231 cells ($1\times10^4$ per well) were seeded in 96-well plates for 24 h before live cell imaging experiments. All media were removed from wells and the cells were treated with RuB3 (0.1 μM) for 2 h. For light treatment, RuB3 was first incubated for 2 h, followed by the 525 nm light irradiation for 10 minutes, and subsequently added the dihydrorhodamine123 (10 μM)
for 30 minutes incubation. The cells were washed with DPBS three times and imaged in serum-free DMEM media. Control wells were treated with DMSO alone (dark/light). The fluorescence images of the ROS generation of RuB3 during PDT treatment on MDA-MB-231 cells were captured on BioTek Cytation 5 Cell Imaging Multi-Mode Reader and the image is analysed by using BioTek Gen5 Software.

**Singlet Oxygen Detection**

MDA-MB-231 cells ($1\times10^4$ per well) were seeded in 96-well plates for 24 h before the live cell imaging experiments. All media were discarded from wells and the cells were treated with RuB3 (0.05 μM) for 2 h. After 2 h incubation, the cells were stained with the BioTracker™ Si-DMA Singlet Oxygen Live Cell Dye, and continue incubated for 45 minutes. For light treatment, the cells were irradiated with 525 nm light source for 10 minutes. Cells were then rinsed with DPBS for three times and cell imaging was done in serum-free DMEM media, visualised by BioTek Cytation 5 Cell Imaging Multi-Mode Reader. The fluorescence intensity was analysed by using BioTek Gen5 Software.

**Cell Apoptosis Assay**

MDA-MB-231 cells were cultured in 6-well plates ($6\times10^4$ per well) for 24 h. The dark plates were incubated with RuB3 (0.1 μM and 0.5 μM) for 24 h, whereas the light plates were first incubated with RuB3 for 2 h, then exposed to the 525 nm light for 10 min, then continue incubated for 24 h. The cells were harvested after the incubation period and washed with cold PBS. The collected cells were resuspended in PBS containing Annexin V-FITC and PI in the dark environment, according to the manufacturer's instructions (Invitrogen). Finally, the apoptosis was detected by flow cytometry.

**Subcellular Localization Studies**

MDA-MB-231 cells ($3\times10^4$ per well) were seeded in the 8-chamber slide. After 24 h of incubation, the slide was incubated with 0.1 μM of RuB3, co-incubated with MitoTracker™ Green FM for 30 minutes. After that, the cells were rinsed twice with DPBS and the LysoTracker™ Red DND-99 (Invitrogen) was added into the cells for lysosome labelling. After 1h of incubation, the cells were washed with DPBS twice and DAPI was added into the cells for mitochondria labelling and incubated for 30 minutes. The cells were washed with PBS twice, fixed with 4% paraformaldehyde for 20 minutes, and then permeabilized with 0.3% Triton X-100 for 15 minutes. Cell imaging was done by using Zeiss Axio Imager Z1 with a 63× oil objective lens. Fluorescence images were collected in the following channels: MitoTracker™ Green FM ($\lambda_{ex}$ = 490 nm, $\lambda_{em}$ = 516 nm); LysoTracker™ Red DND-99 ($\lambda_{ex}$ = 577 nm, $\lambda_{em}$ = 590 nm); DAPI ($\lambda_{ex}$ = 360 nm, $\lambda_{em}$ = 460 nm).

**Intracellular Superoxide Detection with Dihydroethidium (DHE)**

MDA-MB-231 cells (1 × 10^4 cells per well) were seeded in 96-well plates and cultured for 24 h prior to live-cell imaging. The culture medium was then removed, and the cells were incubated with RuB3 at the indicated concentrations (0.01 and 0.05 μM) for 2 h. For the light groups, cells were irradiated with a 525 nm light source for 10 min after the incubation period, whereas the dark groups were kept under the same conditions without irradiation. After treatment, the cells were incubated with dihydroethidium (DHE) according to the manufacturer's recommended protocol for superoxide

detection. The cells were then washed with DPBS three times and imaged in serum-free DMEM medium. Fluorescence images were acquired using a BioTek Cytation 5 Cell Imaging Multi-Mode Reader. The fluorescence intensity was analyzed using BioTek Gen5 software.

**Intracellular Hydroxyl-Radical-Associated ROS Detection with Hydroxyphenyl Fluorescein (HPF)**

MDA-MB-231 cells (1 × 10^4 cells per well) were seeded in 96-well plates and cultured for 24 h before the experiment. The cells were then treated with RuB3 at the indicated concentrations (0.01 and 0.05 μM) for 2 h. For the light groups, cells were exposed to 525 nm irradiation for 10 min, while the dark groups were maintained under identical conditions without light exposure. After treatment, the cells were incubated with hydroxyphenyl fluorescein (HPF) following the manufacturer's protocol to probe hydroxyl-radical-associated oxidative species. The cells were washed three times with DPBS and imaged in serum-free DMEM medium. Fluorescence images were recorded using a BioTek Cytation 5 Cell Imaging Multi-Mode Reader, and fluorescence intensity was quantified using BioTek Gen5 software.

**Measurement of Mitochondrial Respiration**

Mitochondrial respiration was analysed by using Seahorse XF Cell Mito Stress Test Kit (Agilent Technologies, USA) in a Seahorse XFe24 Analyzer (Seahorse Bioscience, USA) according to the manufacturer's specifications. The effects of RuB3 on several mitochondrial respiration parameters (basal and maximal respiration, proton leak, ATP-linked respiration and non-mitochondrial oxygen consumption) were obtained. The effects of RuB3 under various stressors [1 μM oligomycin, 1.5 μM carbonilcyanide p-triflouromethoxyphenylhydra zone (FCCP), and 0.5 μM rotenone/antimycin A] were tested. MDA-MB-231 cells were seeded at a density of $1\times10^4$ cells/well, and treated with 0.05 μM of RuB3 for 2 hours. Cultivated cells were washed twice and kept in an XF assay medium upon measurement. The cells were incubated at 37°C for 30-60 min in a non-$CO_2$ incubator before measurement. The OCR rate was calculated and expressed as pmol/min. Data were analysed using Seahorse Wave software.

**Nanolive Live Cell Imaging**

MDA-MB-231 cells ($1\times10^4$ per well) were seeded on the μ-Dish 35-mm-high (ibidi) plates for 24 hours. After that, the cells were incubated with 0.01 μM of RuB3 and the live cell imaging was observed with the Nanolive 3D Cell Explorer microscope with an excitation wavelength at 525nm.

**Western blot analysis**

Cells were seeded in culture dishes and allowed to attach overnight. The cells were then treated with RuB3 at the indicated concentrations (10 and 50 nM) and incubated under standard culture conditions. For the light-irradiated groups, cells were exposed to light after RuB3 treatment, whereas the dark groups were kept protected from light. After treatment, cells were washed with cold PBS and lysed using RIPA lysis buffer containing protease inhibitor cocktail. The lysates were collected and centrifuged to remove cell debris, and the protein concentration was determined using a BCA protein assay. Equal amounts of protein were separated by SDS-PAGE and transferred onto PVDF membranes. The membranes were blocked with 5% non-fat milk in TBST and then incubated overnight at 4 °C with primary antibodies against Bcl-2, Bax, cleaved caspase-9, cleaved caspase-3, cleaved-PARP, and β-actin. After washing with TBST, the membranes were incubated with the corresponding HRP-conjugated secondary antibodies at room temperature. Protein bands were visualized using an enhanced chemiluminescence detection reagent and imaged using a chemiluminescence imaging system. β-Actin was used as the internal loading control.

**Stability of RuB3 in Biologically Relevant Medium**

The stability of RuB3 in biologically relevant medium was evaluated by UV–vis absorption spectroscopy. RuB3 was dissolved in DMEM/F-12 medium containing 10% FBS to give a final concentration of 5 μM and incubated at 37 °C under dark conditions. At predetermined time points (0, 1, 2, 4, 8, and 24 h), aliquots were collected and their UV–vis spectra were recorded over the range of 200–800 nm using a ChromTech CT-2200 spectrophotometer. The absorbance of the characteristic band of RuB3 was monitored over time, and the relative absorbance was normalized to the value at 0 h. Stability was assessed from changes in the spectral profile, λmax position, and absorbance intensity during incubation.

**Fluorescence imaging of intracellular singlet oxygen generation and photocytotoxicity under normoxic and hypoxic conditions.** Cells were seeded in confocal dishes or multiwell plates and incubated overnight to allow cell attachment. The cells were then treated with RuB3 at the indicated concentration and maintained under controlled normoxic or hypoxic conditions using the gas controller of a BioTek imaging system. Normoxic conditions were set at 21% $O_2$ and 5% $CO_2$, while hypoxic conditions were set at 1% $O_2$ and 5% $CO_2$, with $N_2$ used as the balance gas. For the hypoxia group, the oxygen level was reduced and stabilized before RuB3 treatment and maintained throughout RuB3 incubation, light irradiation, and imaging. For intracellular singlet oxygen detection, cells were incubated with a singlet oxygen fluorescent probe under the corresponding oxygen conditions. After probe loading, the cells were washed with PBS to remove excess probe and then exposed to light irradiation while maintaining the controlled gas environment. The nuclei were counterstained with DAPI. Fluorescence images were acquired using the BioTek imaging system. Red fluorescence was

used to monitor intracellular singlet oxygen generation, and blue fluorescence indicated DAPI-stained nuclei. For photocytotoxicity imaging, cells were treated with RuB3 and irradiated under normoxic or hypoxic conditions as described above. After irradiation, the cells were stained with a live/dead cytotoxicity fluorescent probe according to the manufacturer's protocol. After washing with PBS, fluorescence and bright-field images were acquired using the BioTek imaging system. Cyan/green fluorescence indicated damaged or dead cells, while DAPI staining was used to visualize the nuclei. Bright-field images were collected and merged with fluorescence channels to show cellular morphology and staining distribution.

**Table S1:** Comparison of representative Ru–BODIPY photosensitizers reported in the literature with RuB3 in this work, highlighting differences in ruthenium scaffold, BODIPY design, biological model, reported photodynamic features, and mechanistic focus. This comparison clarifies that the novelty of RuB3 lies not in the general Ru–BODIPY concept, but in its 3-styryl-extended BODIPY–bipyridyl/$[Ru(dmb)_2]^{2+}$ architecture and its TNBC-focused, mitochondria-associated light-triggered apoptotic mechanism.

| System | Ru scaffold | BODIPY design | Main biological model | Main novelty | Difference from RuB3 |
|---|---|---|---|---|---|
| 1 | Ru-bis-terpyridine | BODIPY pendant, biotin targeting | HeLa/HPL1D | High PI, type I/II ROS | Terpyridine/biotin system; not $[Ru(dmb)_2]^{2+}$; not TNBC mitochondrial respiration study |
| 2 | Ru-arene | py-BODIPY labile ligand | Photochemical ligand dissociation | Photoactivated metallodrug | Mechanism is ligand dissociation/DNA binding, not mitochondrial PDT in TNBC |
| 3 | Ru-bis-terpyridine | BODIPY conjugates | TTA upconversion | Ultra-long triplet lifetime | Photophysics/upconversion, not anticancer PDT mechanism |
| 4 | Ru-terpyridine | BODIPY-biotin dyad | HeLa/H1299 spheroids | Biotin targeting, lysosome imaging | Biotin/terpyridine system; different scaffold and biological emphasis |
| 5 | Ru-arene | meso-pyridine BODIPY | HeLa/MDA-MB-231 | PDT activity, but dark toxicity/aggregation concerns | Lacks detailed intracellular ROS/apoptosis/mitochondrial respiration mechanism |
| This work | $[Ru(dmb)_2]^{2+}$ | 3-styryl-extended BODIPY–bipyridyl | MDA-MB-231 TNBC | Mitochondria-associated ROS, Seahorse OCR, apoptosis, Nanolive | TNBC-focused mitochondrial photodynamic mechanism |

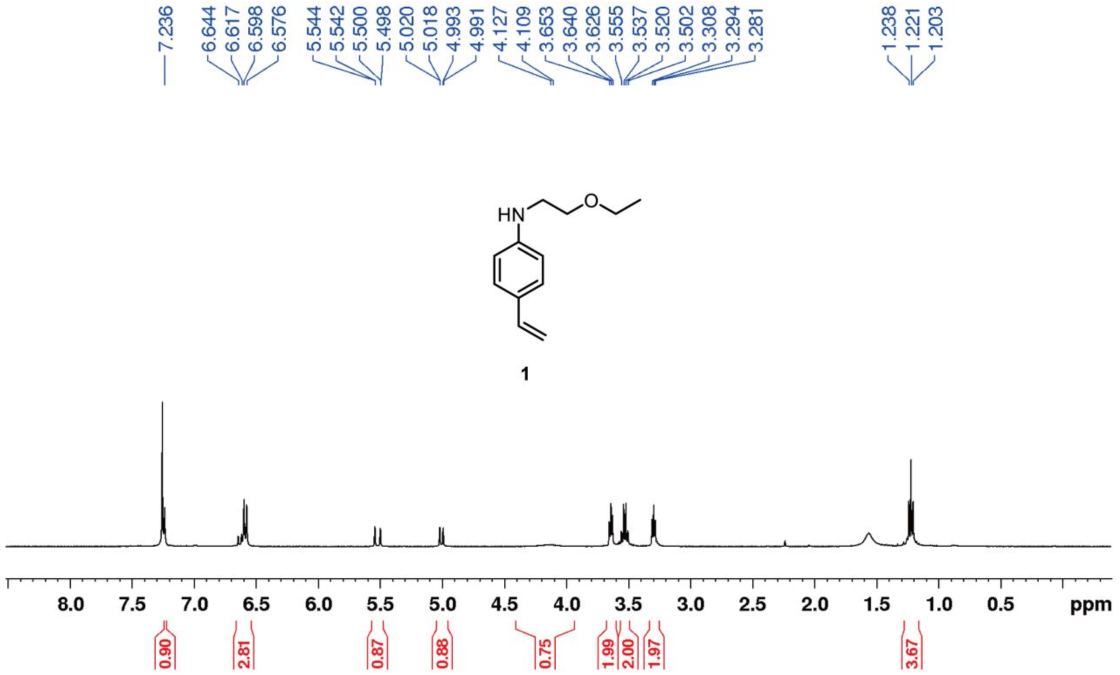


**Figure S1.** $^1$H-NMR spectrum of **1**. (400 MHz, 298K, $CDCl_3$)

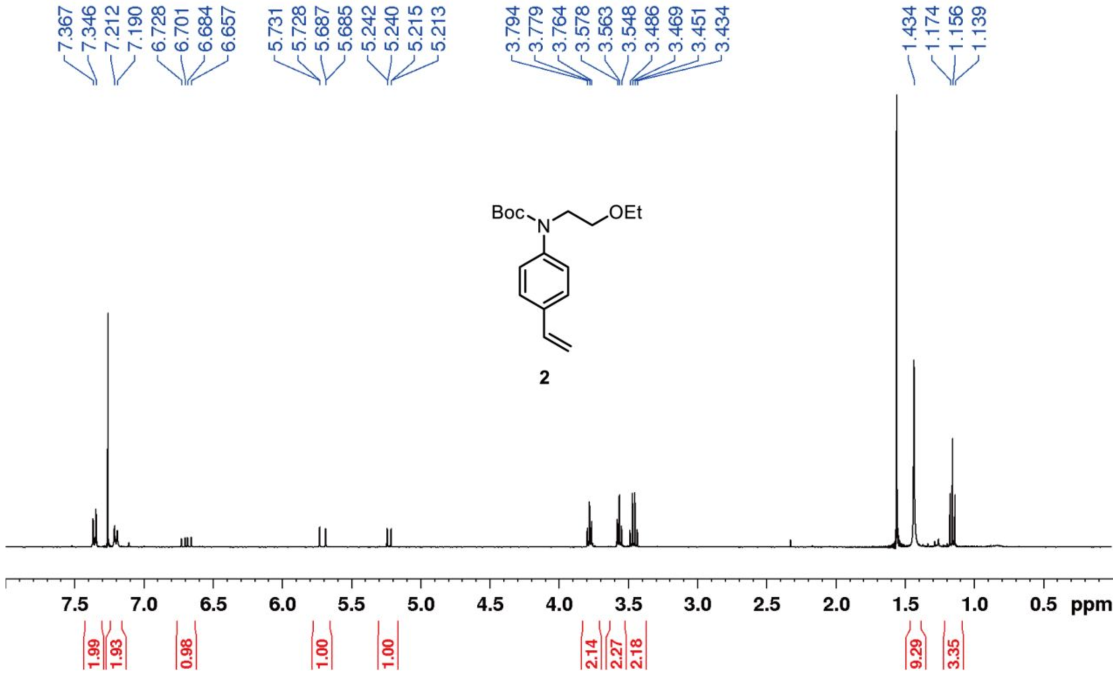


**Figure S2.** $^1$H-NMR spectrum of **2**. (400 MHz, 298K, $CDCl_3$)

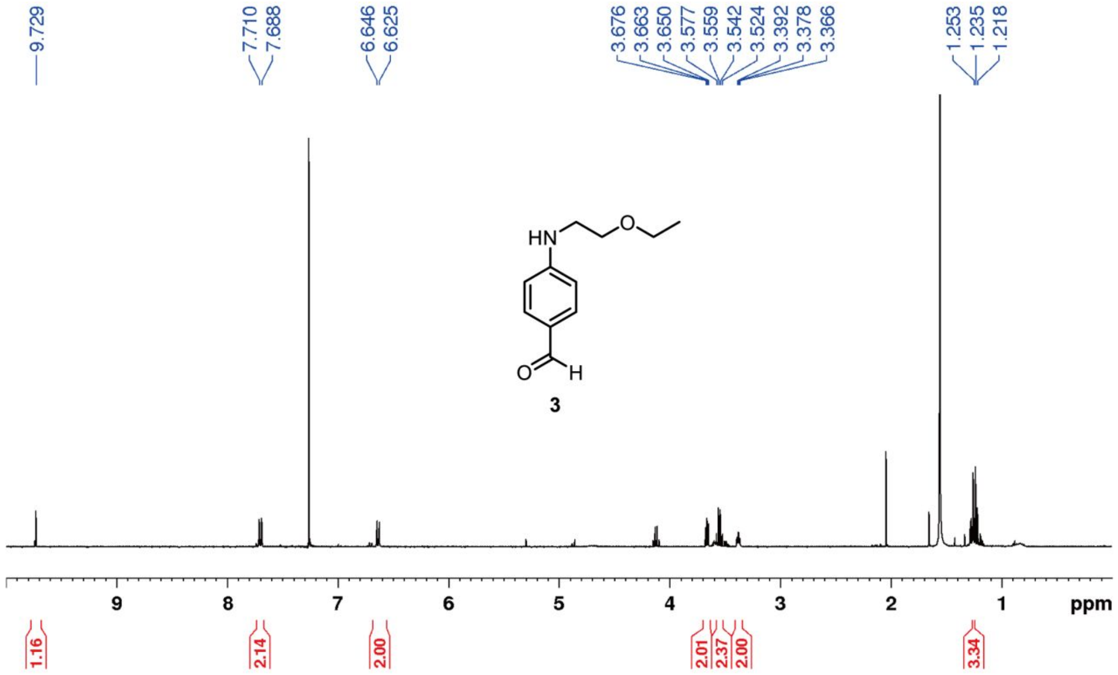


**Figure S3.** $^{1}$H-NMR spectrum of **3**. (400 MHz, 298K, $CDCl_3$)

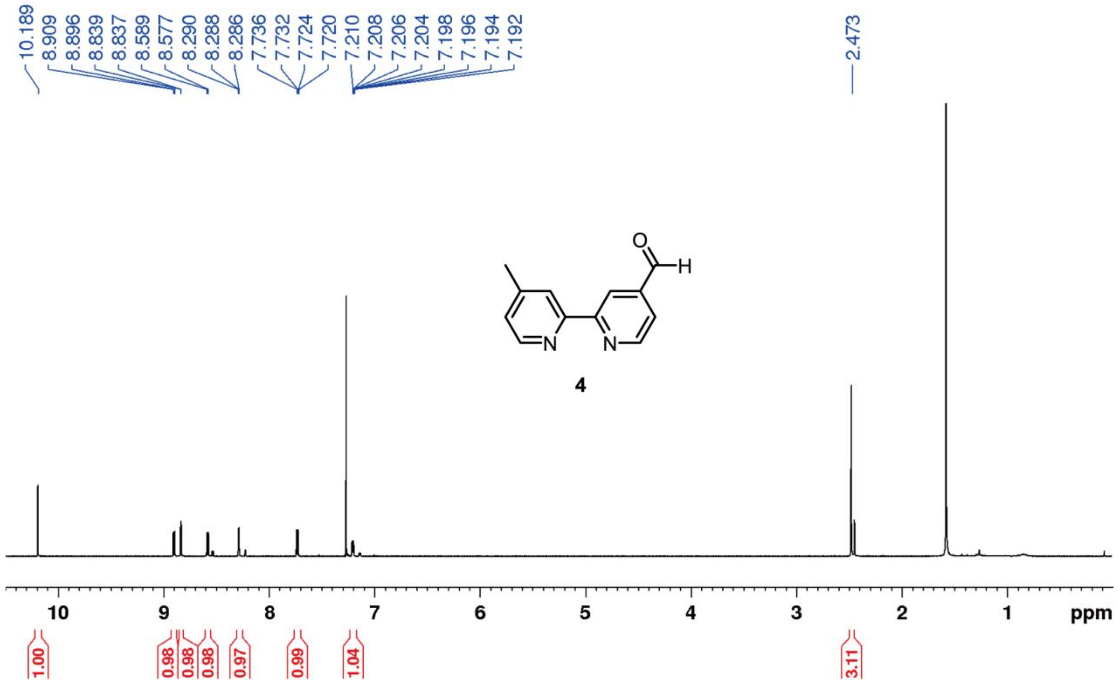


**Figure S4.** $^{1}$H-NMR spectrum of **4**. (400 MHz, 298K, $CDCl_3$)

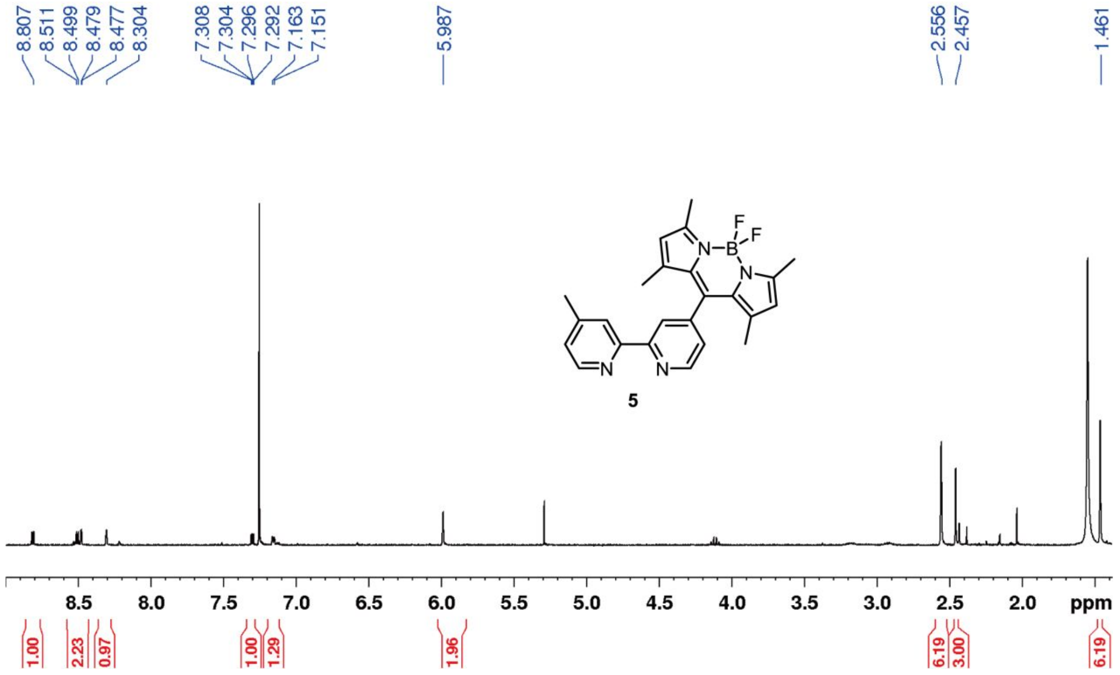


**Figure S5.** $^{1}$H-NMR spectrum of **5**. (400 MHz, 298K, $CDCl_3$)

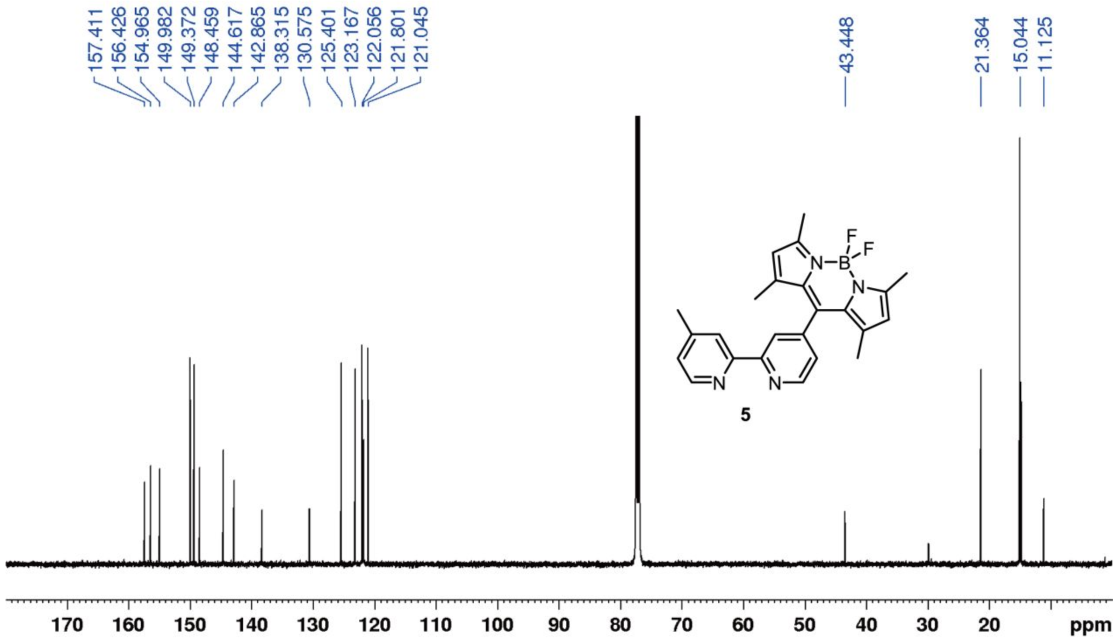


**Figure S6.** $^{13}$C NMR (125 MHz, $CDCl_3$) of **5**. (400 MHz, 298K, DMSO-$d_6$)

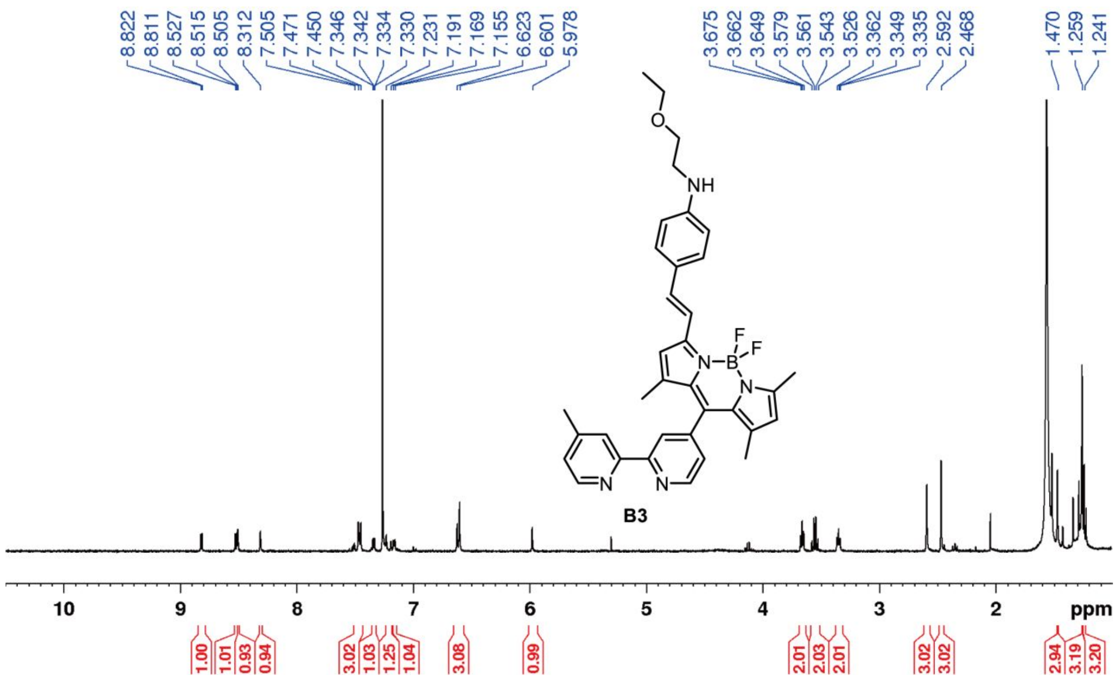

**Figure S7.** $^{1}$H-NMR spectrum of **B3**. (400 MHz, 298K, $CDCl_3$)

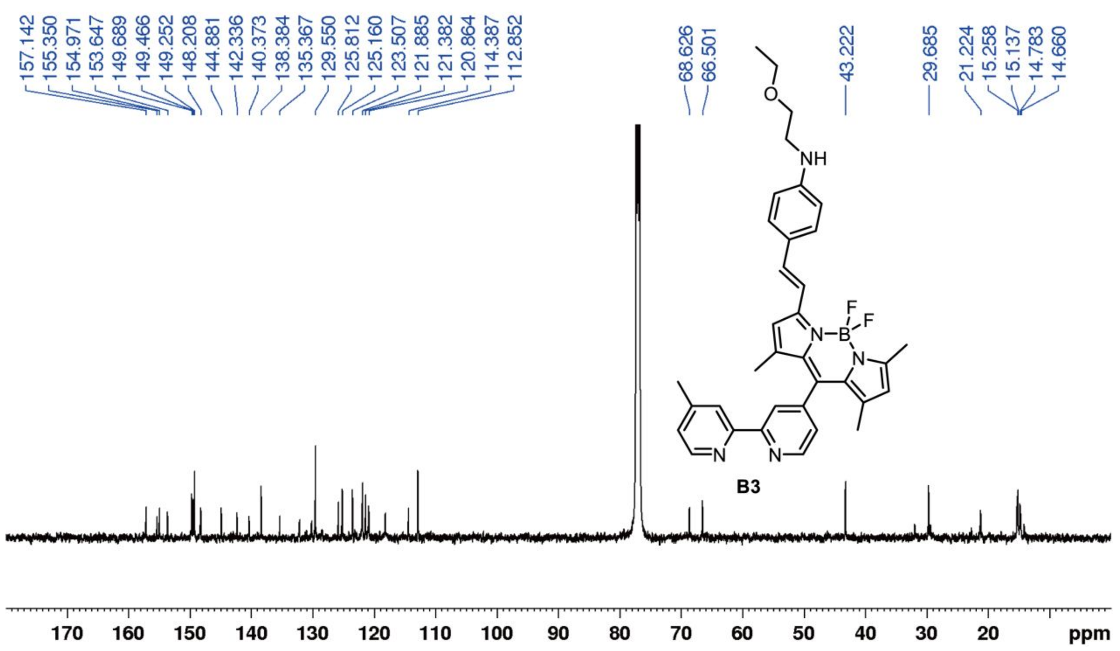

**Figure S8.** $^{13}$C NMR (125 MHz, $CDCl_3$) of **B3**. (400 MHz, 298K, DMSO-$d_6$)

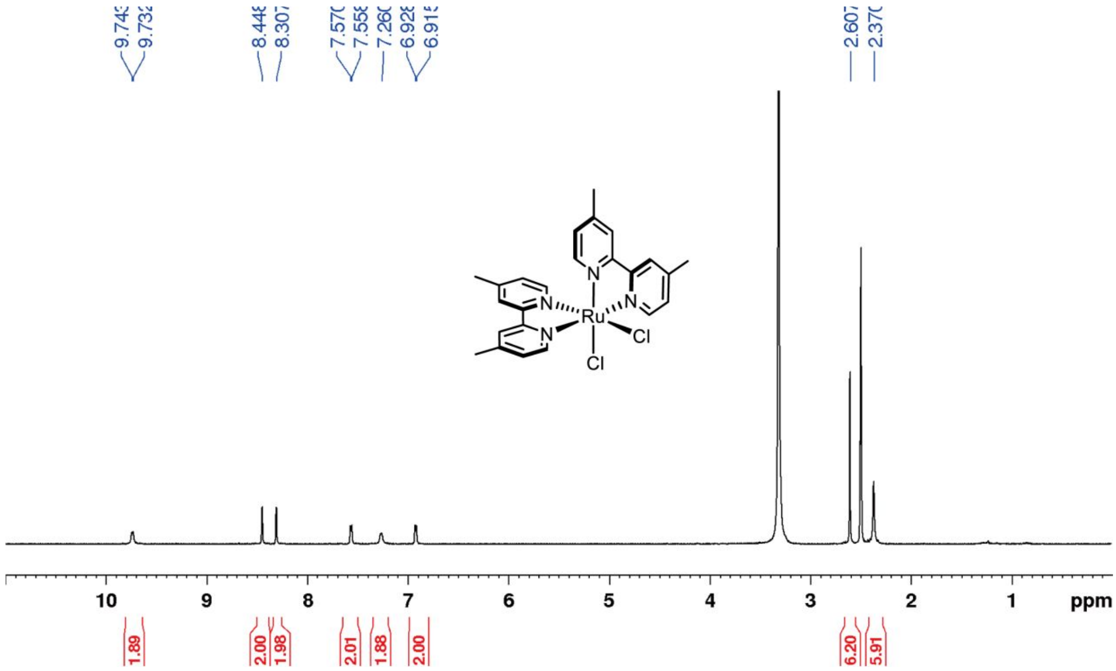


**Figure S9.** ¹H-NMR spectrum of **cis-[Ru(dmb)$_2$Cl$_2$]**. (400 MHz, 298K, DMSO-d$_6$)

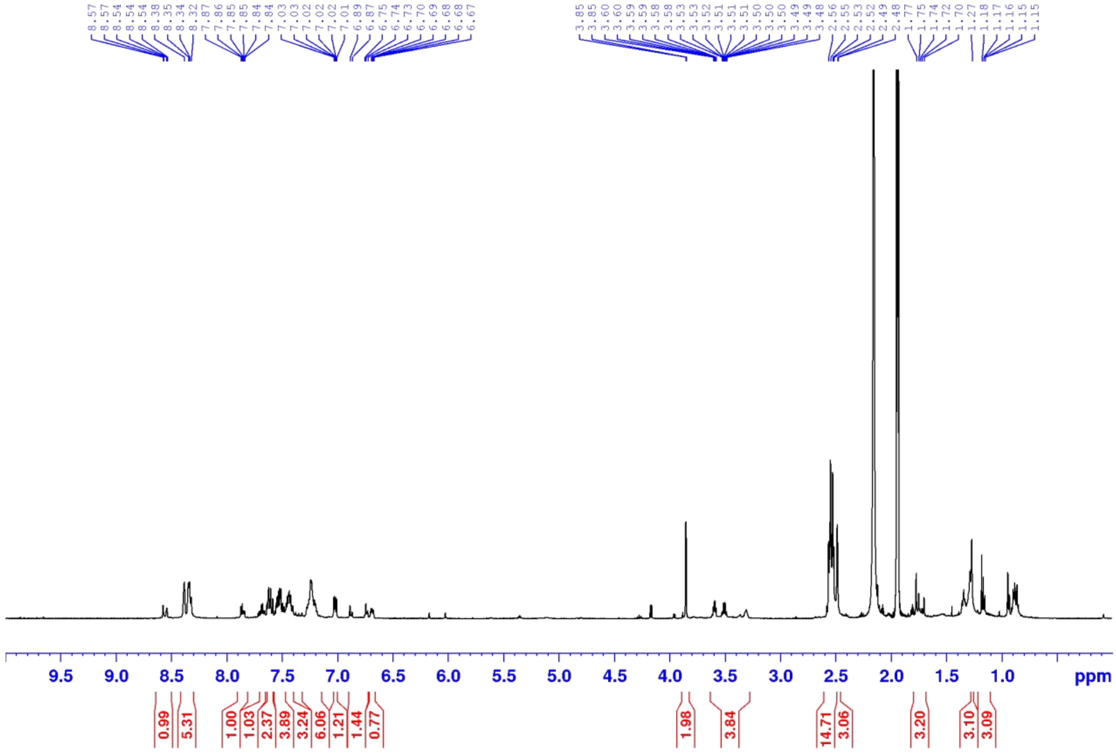

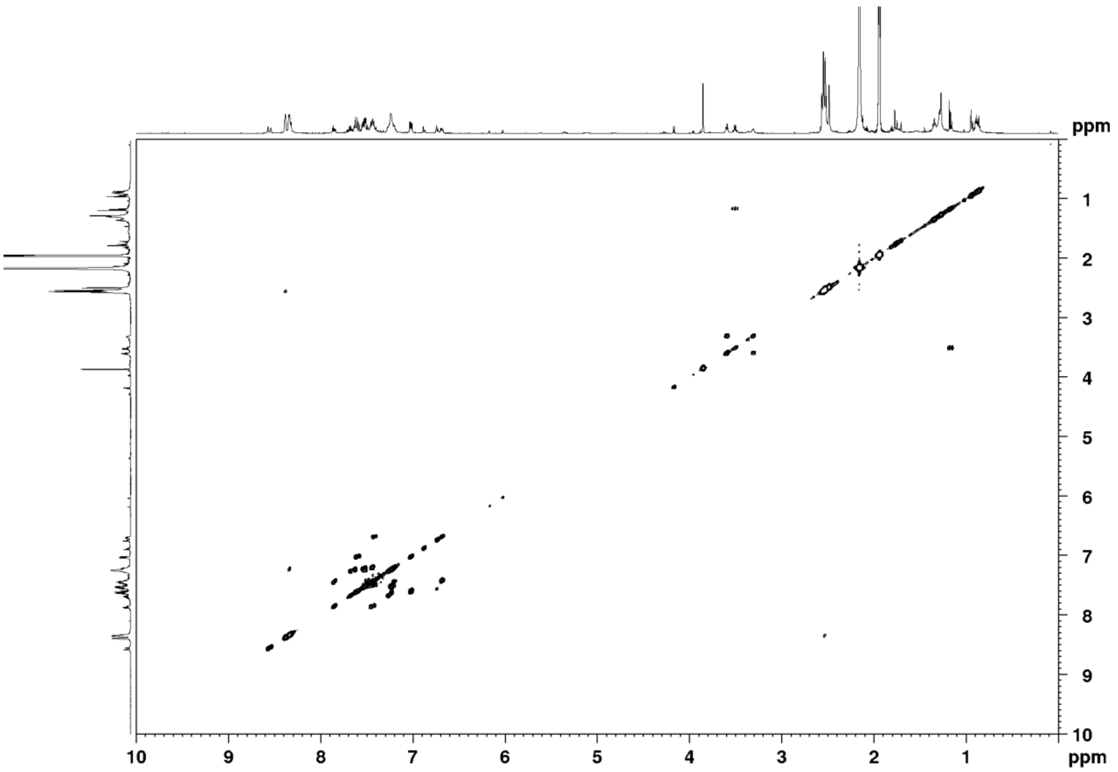


COSY spectrum of **RuB3**. (500 MHz, 298K, $CD_3CN$)

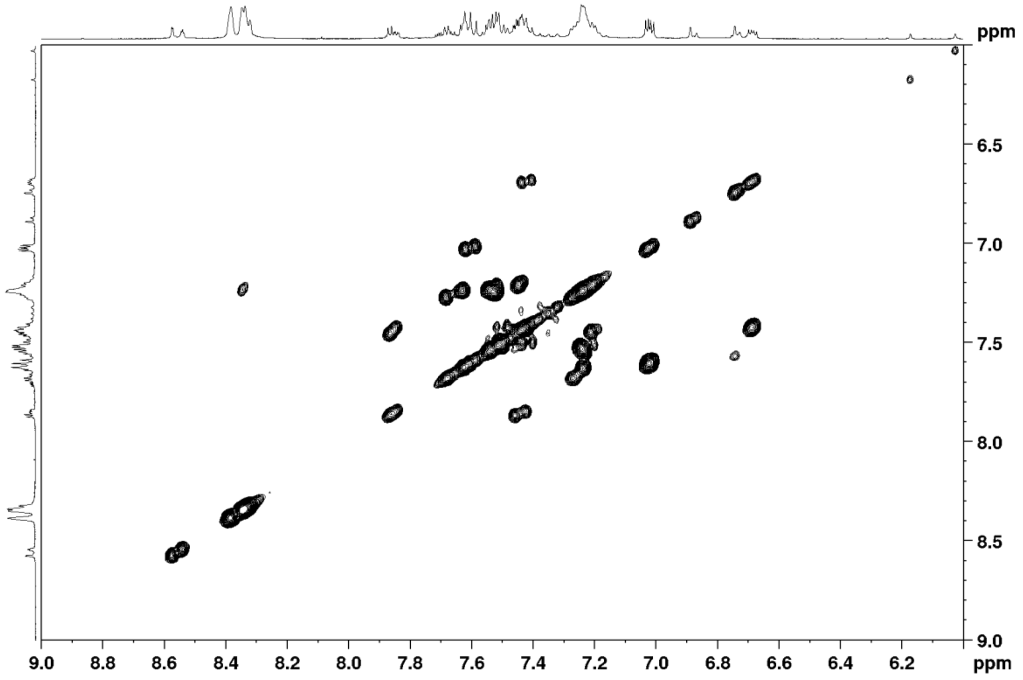


COSY spectrum (aromatic) of **RuB3**. (500 MHz, 298K, $CD_3CN$)

**Figure S10.** $^1$H-NMR spectrum of **RuB3**. (400 MHz, 298K, $CD_3CN$)

Experimental:

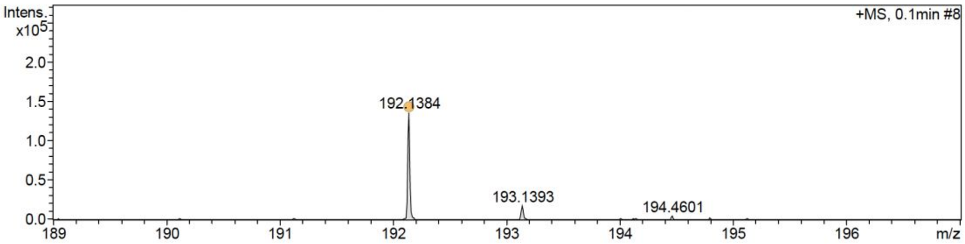


Simulated:

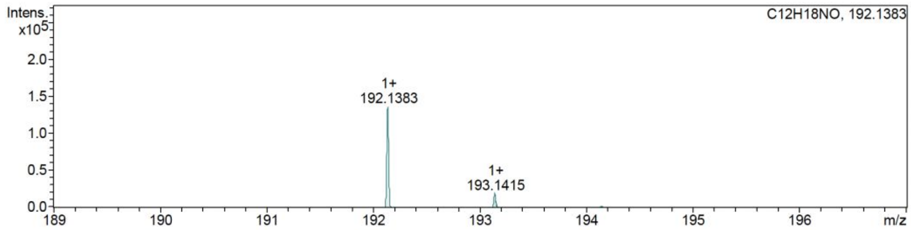


**Figure S11.** High-resolution ESI mass spectrum of compound 1 recorded in methanol, showing the experimental spectrum in the top panel and the simulated isotopic pattern in the bottom panel. The observed peak at m/z 192.1384 agrees well with the calculated value of m/z 192.1383 for $C12H18NO$.

Experimental:

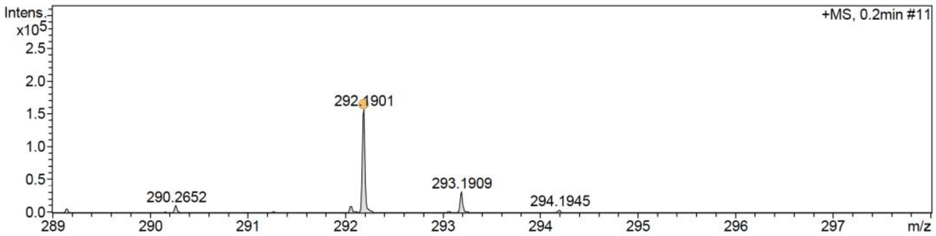


Simulated:

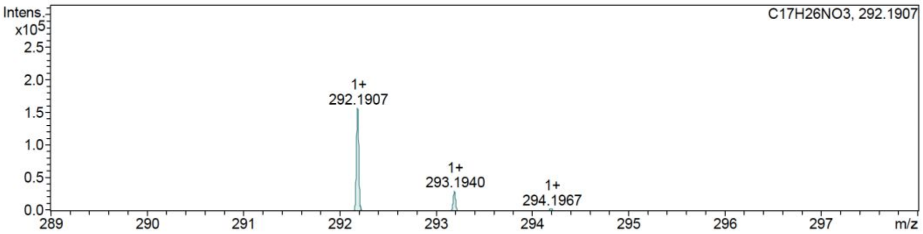


**Figure S12.** High-resolution ESI mass spectrum of compound 2 recorded in methanol, showing the experimental spectrum in the top panel and the simulated isotopic pattern in the bottom panel. The observed peak at m/z 292.1901 agrees well with the calculated value of m/z 292.1907 for $C17H26NO3$.

Experimental:

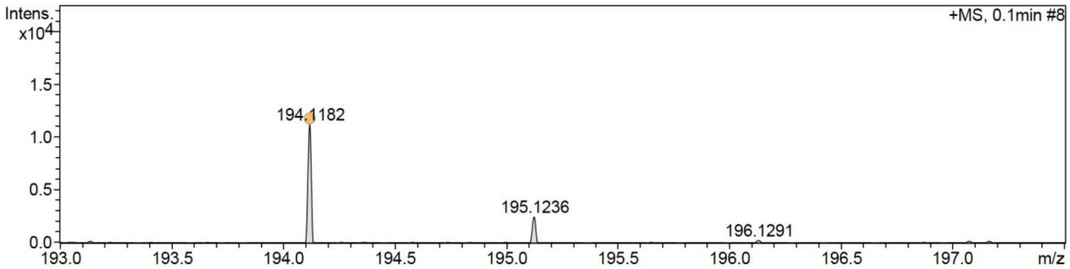


Simulated:

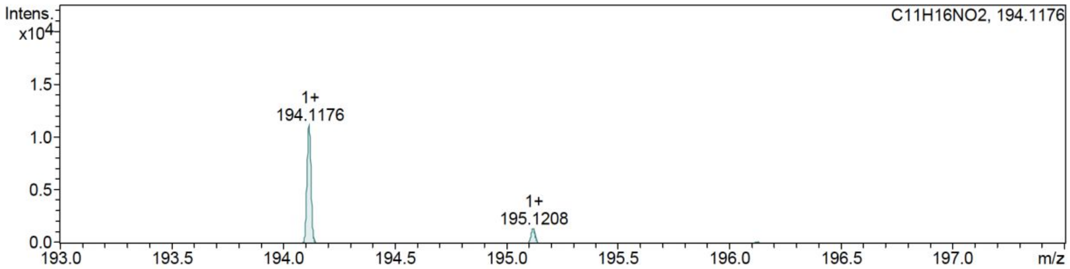


**Figure S13.** High-resolution ESI mass spectrum of compound 3 recorded in methanol, showing the experimental spectrum in the top panel and the simulated isotopic pattern in the bottom panel. The observed peak at m/z 194.1182 agrees well with the calculated value of m/z 194.1176 for C11H16NO2.

Experimental:

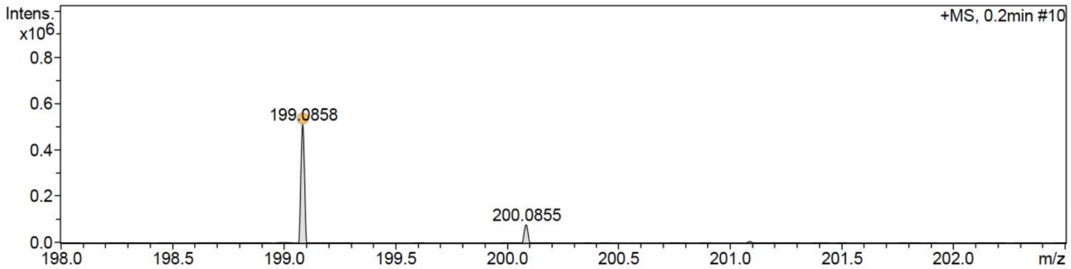


Simulated:

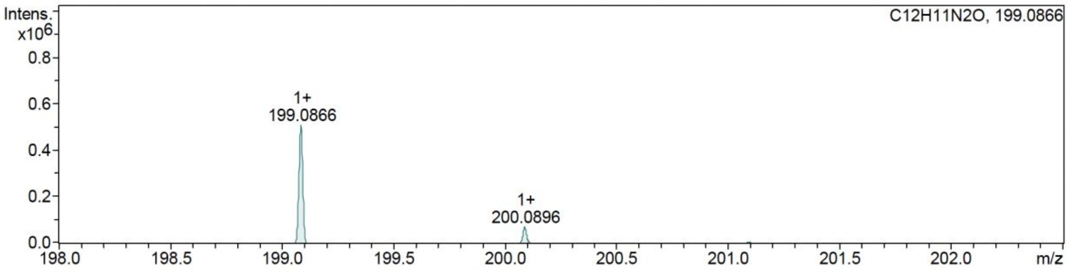


**Figure S14.** High-resolution ESI mass spectrum of compound 4 recorded in methanol, showing the experimental spectrum in the top panel and the simulated isotopic pattern in the bottom panel. The observed peak at m/z 199.0858 agrees well with the calculated value of m/z 199.0866 for C12H11N2O.

Experimental:

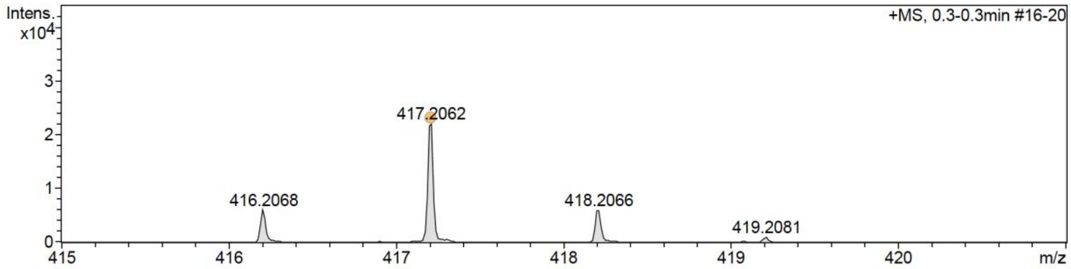


Simulated:

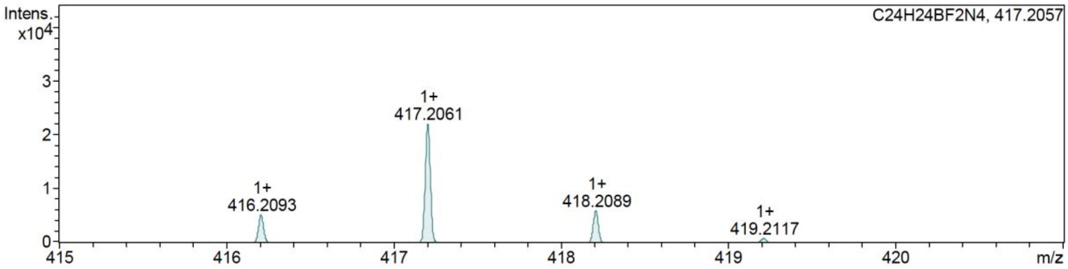


**Figure S15.** High-resolution ESI mass spectrum of compound 5 recorded in methanol, showing the experimental spectrum in the top panel and the simulated isotopic pattern in the bottom panel. The observed peak at m/z 417.2062 agrees well with the calculated value of m/z 417.2061 for C24H24BF2N4

Experimental:

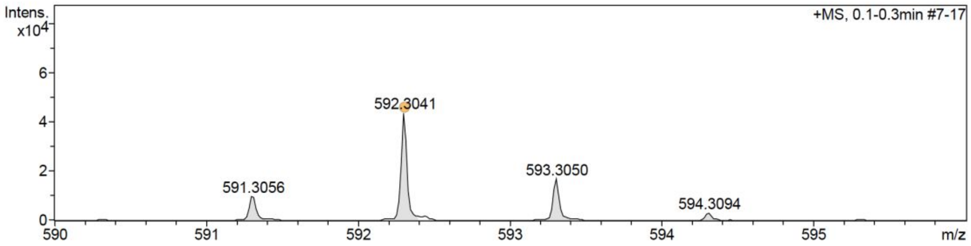


Simulated:

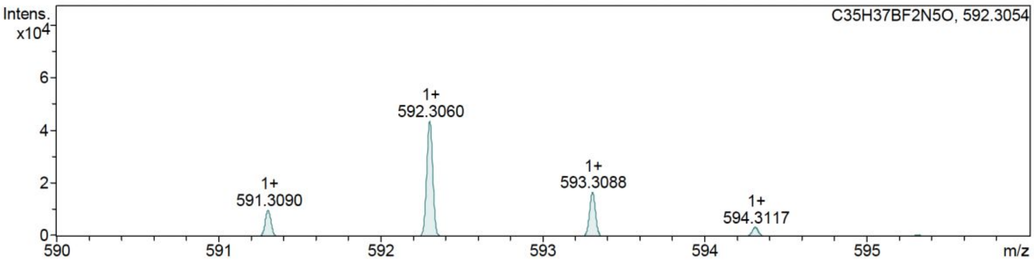


**Figure S16.** High-resolution ESI mass spectrum of compound B3 recorded in methanol, showing the experimental spectrum in the top panel and the simulated isotopic pattern in the bottom panel. The observed peak at m/z 592.3041 agrees well with the calculated value of m/z 592.3060 for C35H37BF2N5O.

Experimental:

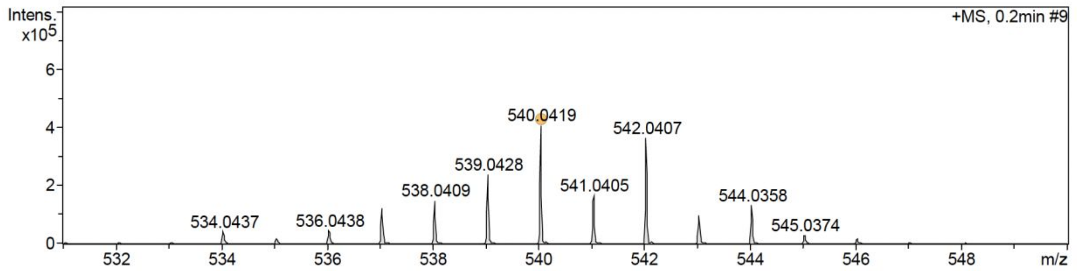


Simulated:

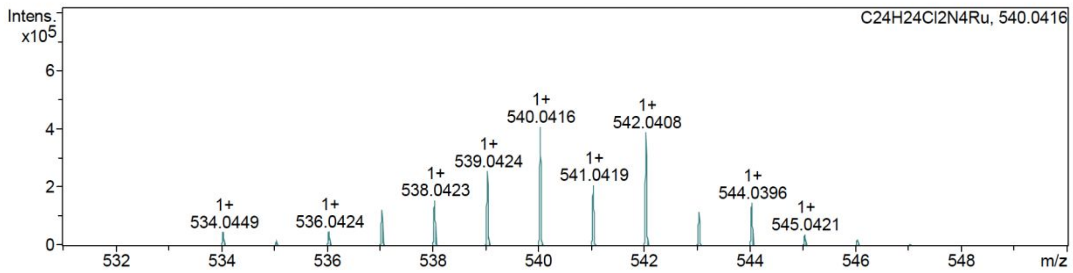


**Figure S17.** High-resolution ESI mass spectrum of cis-[Ru(dmb)$_2$Cl$_2$] recorded in methanol, showing the experimental spectrum in the top panel and the simulated isotopic pattern in the bottom panel. The observed peak at m/z 540.0419 agrees well with the calculated value of m/z 540.0416 for C24H24Cl2N4Ru.

Experimental:

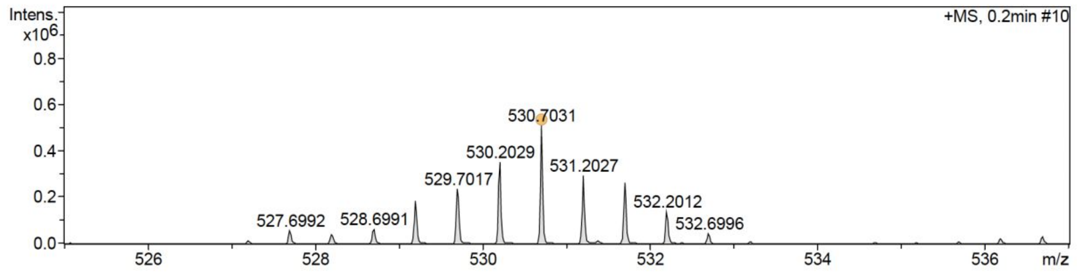


Simulated:

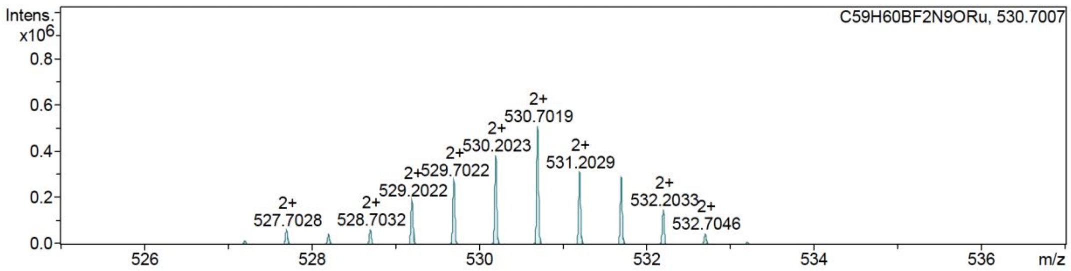


**Figure S18.** High-resolution ESI mass spectrum of RuB3 recorded in methanol, showing the experimental spectrum in the top panel and the simulated isotopic pattern in the bottom panel. The observed peak at m/z 530.7031 agrees well with the calculated value of m/z 530.7019 for C59H60BF2N9ORu as the doubly charged species [M]2+.

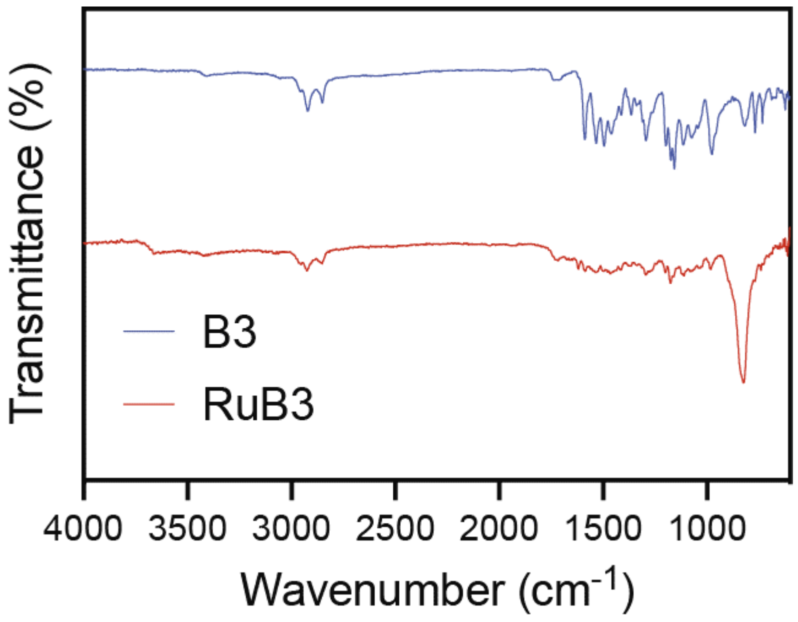


**Figure S19.** The ATR-IR spectrum of **B3** and **RuB3**.

A notable feature is the appearance of a very intense absorption in RuB3 near ~840 $cm^{-1}$, which is absent or much weaker in B3 and is reasonably assigned to the presence of $PF6^{-}$ counterions in the isolated ruthenium complex. This band is therefore consistent with successful isolation of the cationic RuB3 salt as the hexafluorophosphate form.

**Table S2.** Selected transitions for TD-DFT of B3 and RuB3.

| Sample | Observed wavelength (nm) | Calculated wavelength (nm) | Transition | Character |
|---|---|---|---|---|
| B3 | 605 | 602 | HOMO → LUMO | BODIPY Ligand → dmb |
| RuB3 | 445 | 446<br>427 | H-4 → L+1<br>H-4 → LUMO | MLCT (Metal → dmb)<br>MLCT (Metal → BODIPY) |

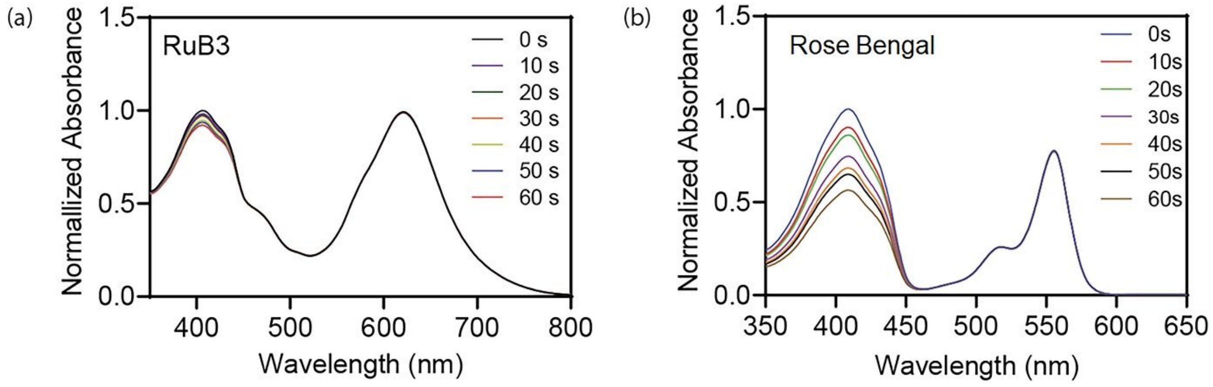


**Figure S20.** Singlet oxygen generation by RuB3, B3, and Rose Bengal monitored using DPBF as a chemical trap under visible-light irradiation. Time-dependent UV–vis absorption spectra of DPBF in the presence of (a) RuB3 and (b) Rose Bengal upon irradiation for 0–60 s. The decrease in DPBF absorbance around 410 nm indicates photoinduced singlet oxygen generation.

**Table S3.** DPBF degradation rate constant (k) and singlet oxygen quantum yield ($\Phi_{\Delta}$) for RuB3 compared to Rose Bengal.

| Sample | $k$ ($min^{-1}$) | $\Phi_{\Delta}$ |
|---|---|---|
| B3 | 0.00183 | 0.07 |
| RuB3 | 0.0791 | 0.12 |
| Rose Bengal | 0.5362 | 0.76 |

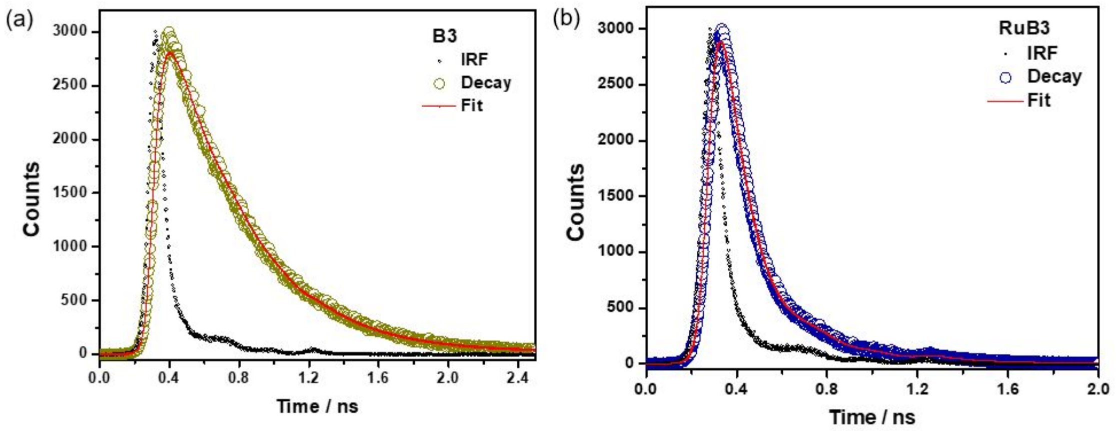


**Figure S21. Time-resolved fluorescence decay profiles of B3 and RuB3.** Fluorescence decay curves of (a) **B3** and (b) **RuB3** recorded under pulsed excitation at 450 nm, showing the instrument response function (IRF, black dots), experimental decay data, and fitted decay curves. B3 exhibits a longer fluorescence decay profile than RuB3, whereas RuB3 shows a markedly shortened excited-state lifetime after coordination to the ruthenium center. This fluorescence quenching indicates that ruthenium coordination significantly modulates the excited-state deactivation pathway of the BODIPY ligand, consistent with enhanced intersystem crossing and improved photosensitization behavior.

**Table S4**. Electrochemical properties of the compound.[a]

| Compound | $E_{p, a1}{}^{c}$ /V | $E_{p, a1}{}^{c}$ /V | $E_{1/2\ red1}{}^{b}$ /V | $E_{p, c1}{}^{c}$ /V | $E_{p, c2}{}^{c}$ /V |
|---|---|---|---|---|---|
| B3 | 0.46 | 0.65 | -1.10 | -0.735 | -1.83 |
| RuB3 | 1.43 | - | -0.083 | -1.37 | -1.83 |

[a] Measured in dichloromethane containing 0.1 M tetra-n-butylammonium perchlorate (TBAP), 1 mM compound, glassy carbon working electrode, and Pt counter electrode and a scan rate of 100 mV/s.

[b] Peak potential for the reversible process.

[c] Peak potential for the irreversible process.

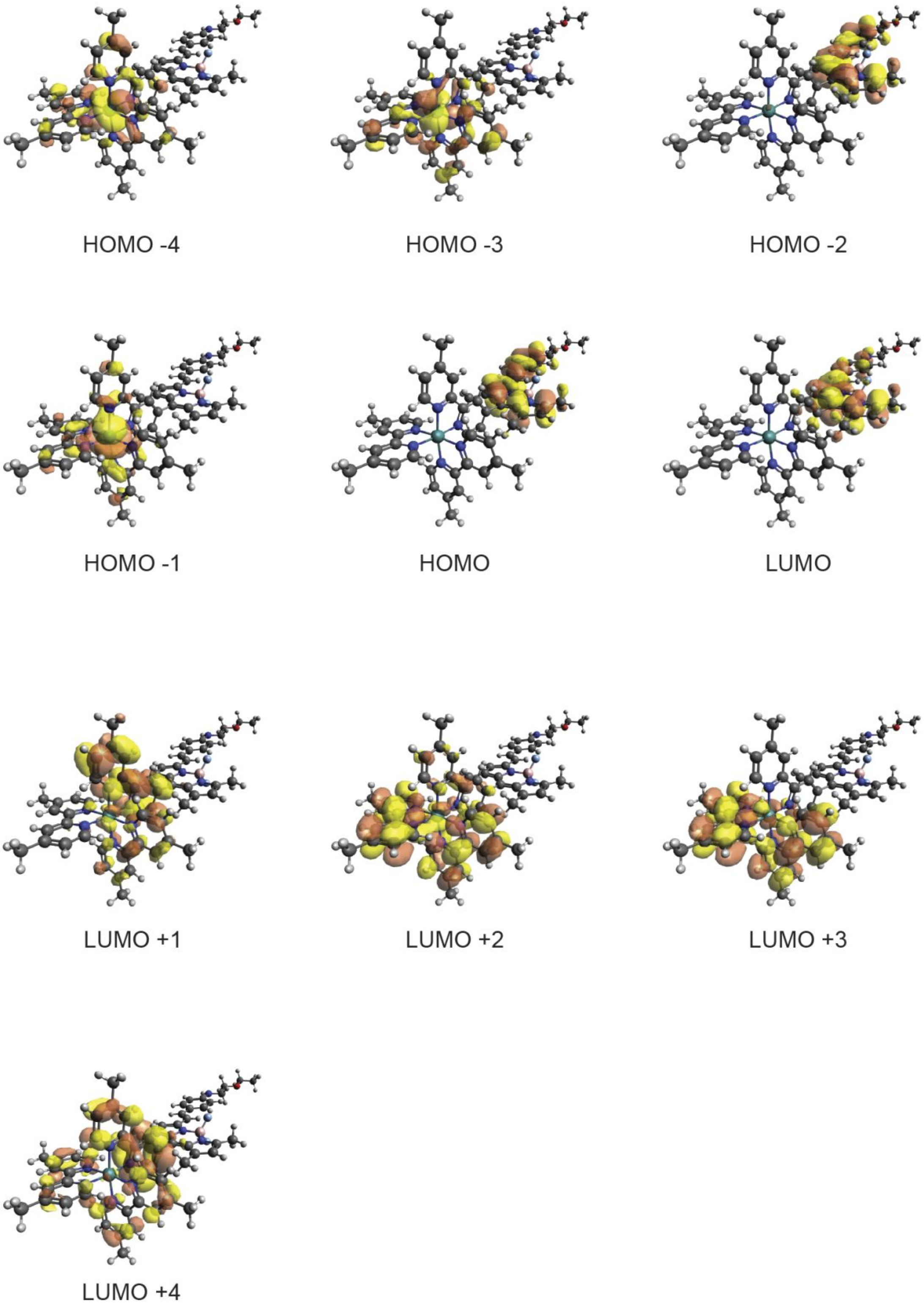


**Figure S22.** Presentation of the orbitals of complex **RuB3** at B3LYP/LanL2DZ level of theory.

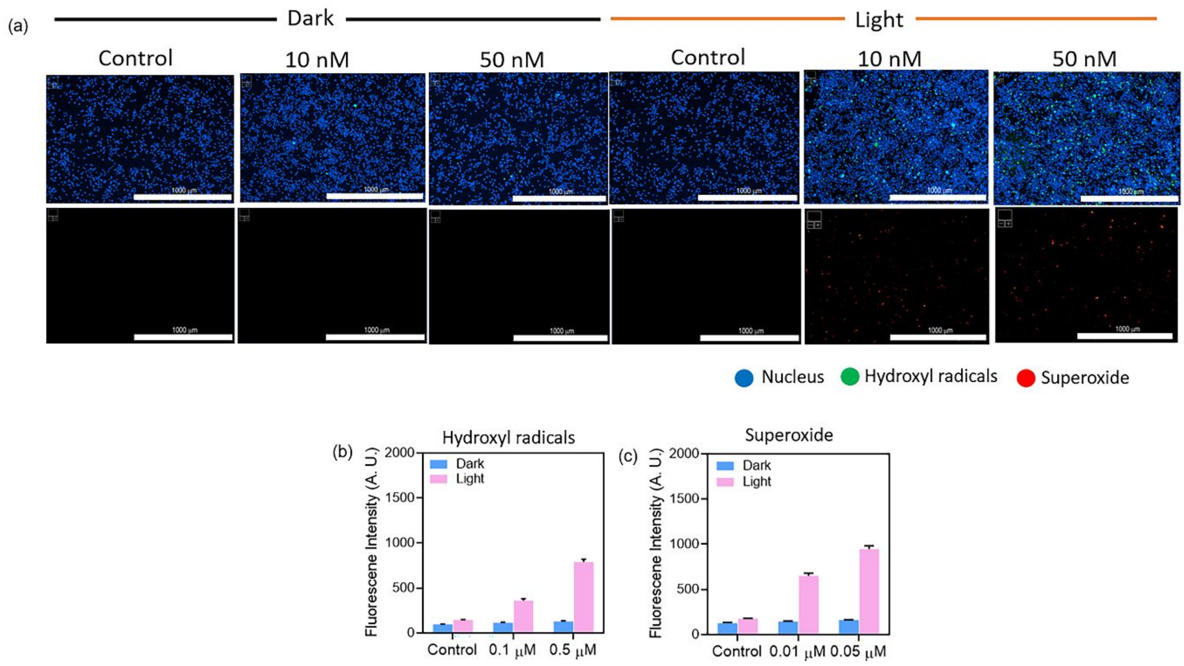


**Figure S23.** Evaluation of Type I ROS-related signals generated by RuB3 in MDA-MB-231 cells under dark and light conditions. (a) Representative fluorescence images show nuclei stained in blue, HPF fluorescence in green, and DHE fluorescence in red at control, 10 nM, and 50 nM RuB3. Minimal fluorescence was observed in the dark groups, whereas light irradiation produced clear, concentration-dependent increases in both HPF and DHE signals. Quantitative fluorescence analysis (A.U.) is shown in (b) hydroxyl radicals generation and (c) superoxide generation. Data are presented as mean ± SD. Scale bar = 1000 μm.

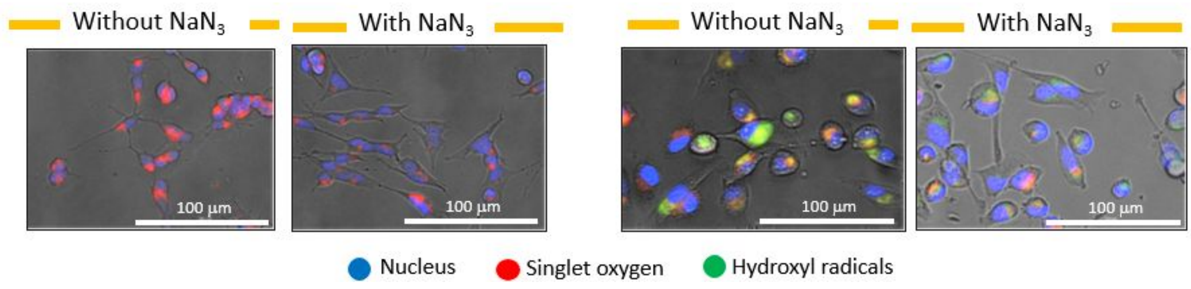


**Figure 24. $NaN_3$ quenching experiment confirming the involvement of singlet oxygen in RuB3-mediated photocytotoxicity.** Representative merged fluorescence microscopy images of cells treated with RuB3 under light irradiation in the absence and presence of $NaN_3$, a singlet oxygen quencher. The red/blue merged images indicate RuB3-related intracellular localization, while the green fluorescence signal reflects hydroxyl radicals generation. Compared with the $NaN_3$-free condition, $NaN_3$ treatment markedly suppresses the red and green fluorescence signal, supporting that RuB3 produces intracellular singlet oxygen upon light activation. Nuclei are counterstained in blue. Scale bars: 100 μm.

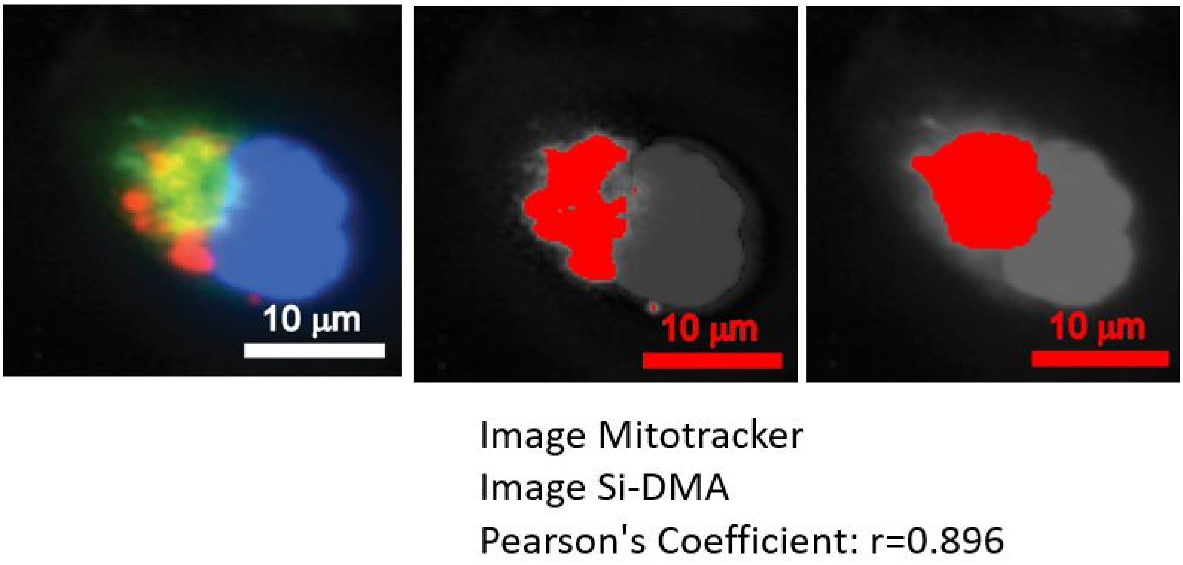


**Figure S25:** Colocalization analysis of mitochondrial-associated singlet oxygen generation in MDA-MB-231 cells using MitoTracker Green and Si-DMA. The merged fluorescence image (left) and the corresponding segmented regions for MitoTracker and Si-DMA signals (middle and right) show substantial spatial overlap. Quantitative analysis gave a Pearson's correlation coefficient of r = 0.896, indicating strong colocalization between the mitochondrial marker and the singlet oxygen probe signal. Scale bar = 10 μm.

**Table S5. Estimated subcellular Ru distribution in MCF-7 cells based on ICP data.**

| **Complex** | **Cytoplasm** | **Mit/Golgi/ER** | **Nucleus** | **Cytoskeleton** |
|---|---|---|---|---|
| MCF-7 | 4.2 ± 0.3 | 12.8 ± 0.4 | 10.6 ± 0.5 | 15.4 ± 0.6 |
| MDA-MB-231 | 5.5 ± 0.4 | 21.6 ± 0.5 | 7.1 ± 0.6 | 4.8 ± 0.5 |
| H184B5F5/M10 cells | 1.0 ± 0.2 | 0.9 ± 0.2 | 1.1 ± 0.2 | 4.8 ± 0.4 |

Ru(ng)/Cell ($x10^6$)

**Table S6. Time-dependent absorption stability of RuB3.** Changes in the maximum absorption wavelength (λ_max), absorbance at λ_max, and relative absorbance of RuB3 were monitored over 24 h in PBS with 10% FBS, fetal bovine serum). RuB3 showed only a slight decrease in absorbance from 0.412 to 0.361, corresponding to 87.6% retention after 24 h, with minimal λ_max shift from 605 to 603 nm. These results indicate good absorption stability of RuB3 under the tested conditions.

| **Time (h)** | λmax (nm) | Absorbance at λmax | Relative absorbance (%) |
|---|---|---|---|
| 0 | 605 | 0.412 | 100 |
| 1 | 605 | 0.408 | 99.0 |
| 2 | 605 | 0.403 | 97.8 |
| 4 | 604 | 0.395 | 95.9 |
| 8 | 604 | 0.387 | 93.9 |
| 24 | 603 | 0.361 | 87.6 |

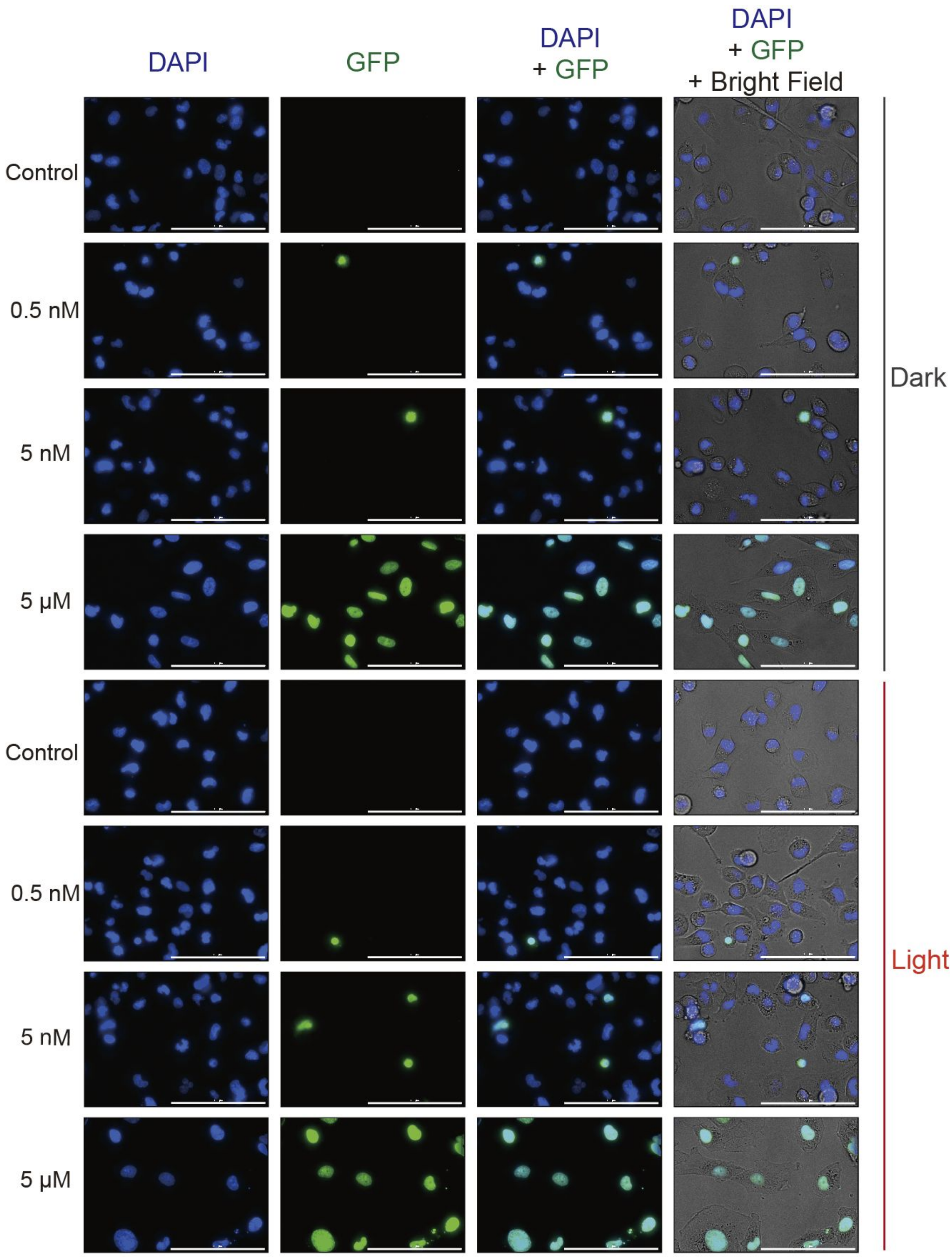


**Figure S26.** Cell Viability test of MDA-MB-231 for 24 h after adding the RuB3 (40× objective). Blue = live cell, green = dead cell.

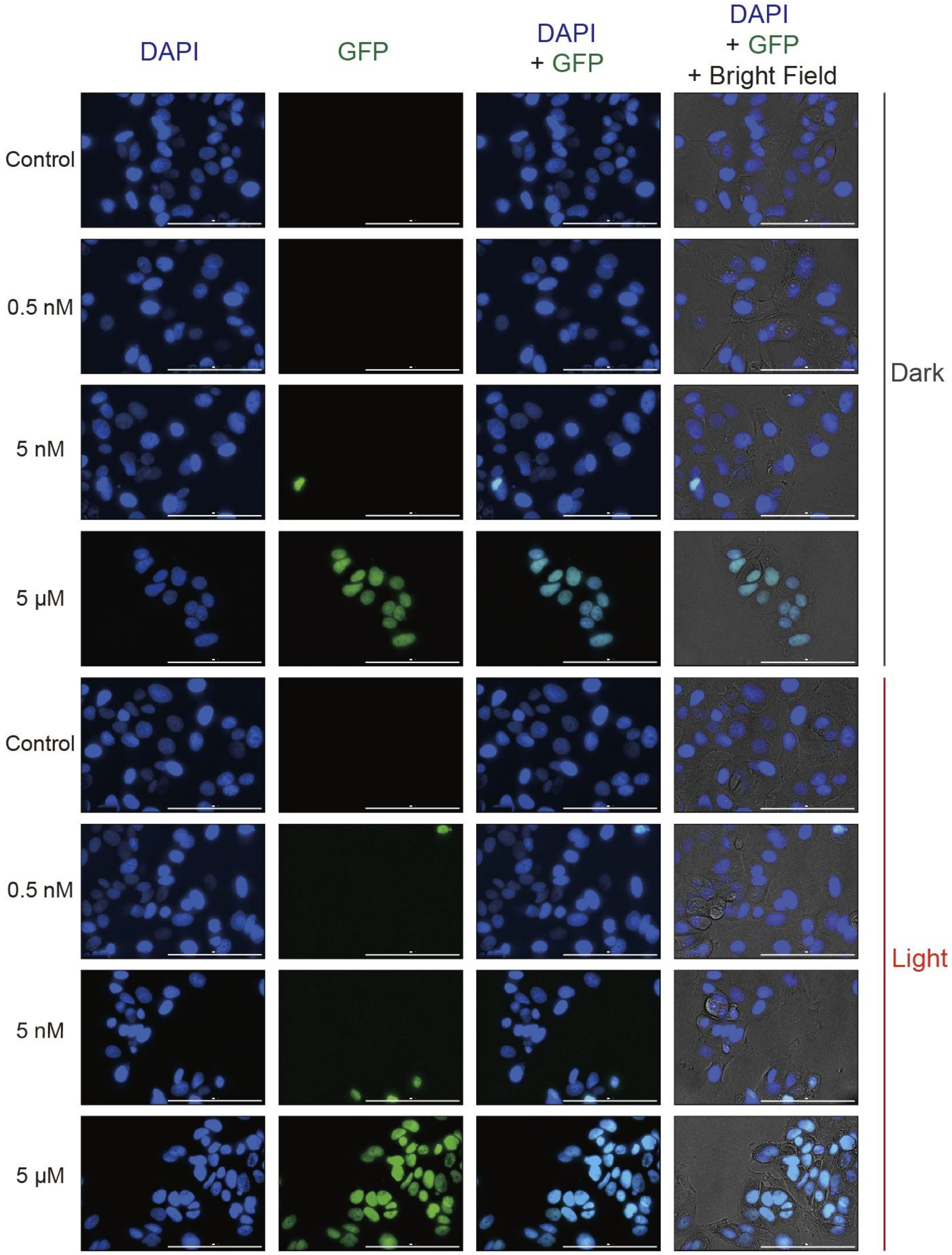


**Figure S27.** Cell Viability test of MCF-7 for 24 h after adding the RuB3 (40× objective). Blue = live cell, green = dead cell.

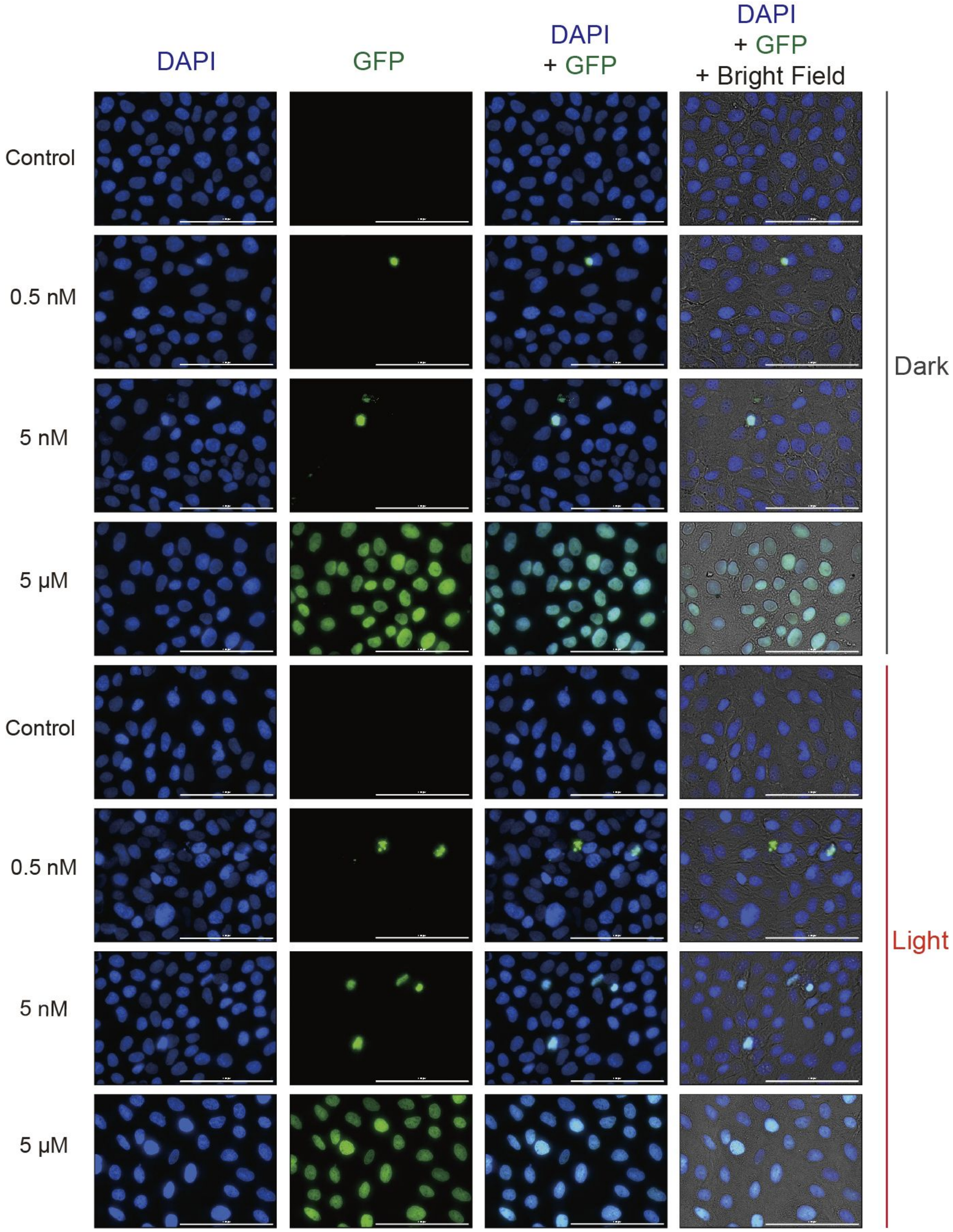


**Figure S28.** Cell Viability test of H184B5F5/M10 for 24 h after adding the RuB3 (40× objective). Blue = live cell, green = dead cell.

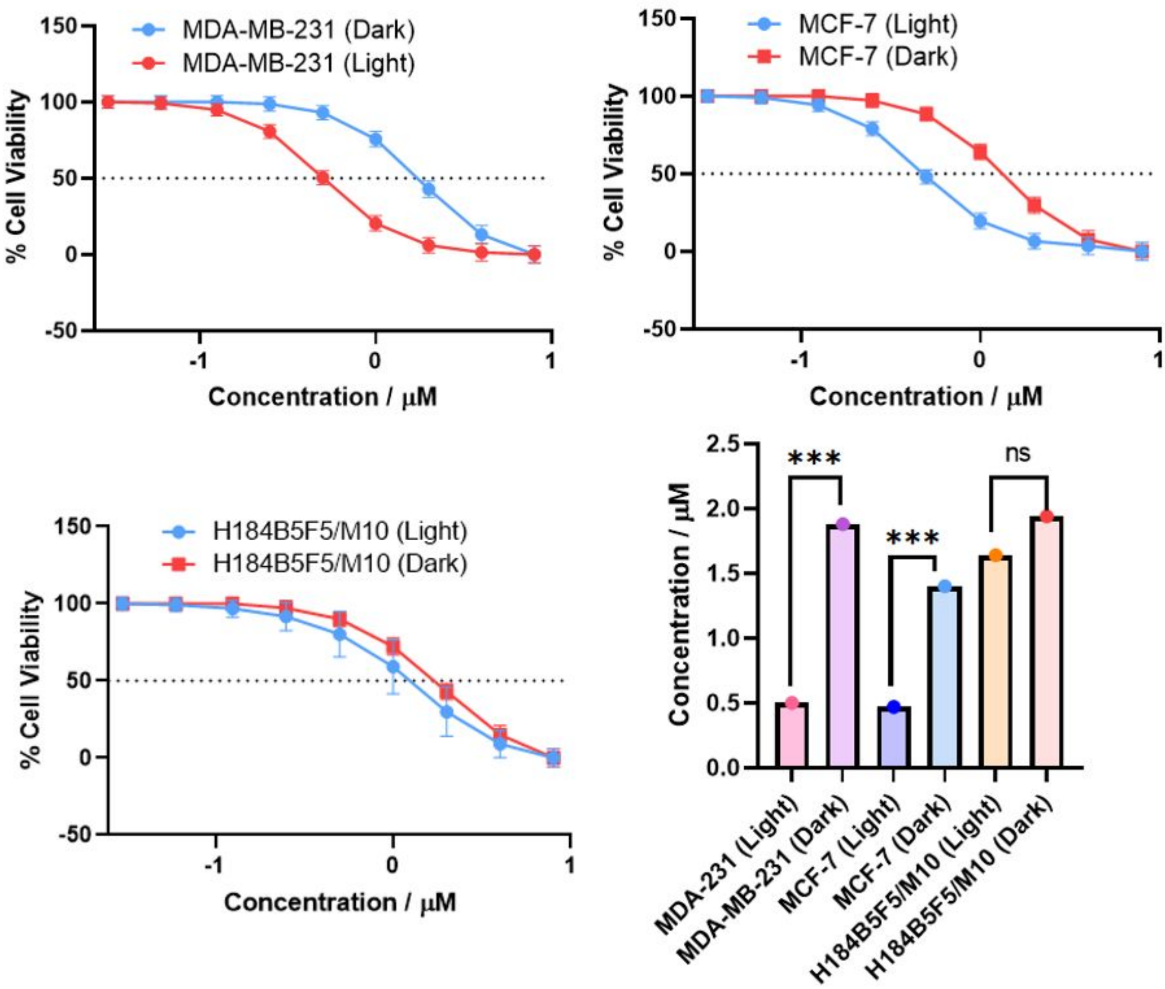


**Figure S29. Photocytotoxicity of RuB3 toward breast cancer and normal breast epithelial cells.** Dose–response cell viability curves of MDA-MB-231, MCF-7, and H184B5F5/M10 cells treated with RuB3 under dark and light-irradiated conditions. The dotted line indicates 50% cell viability for $IC_{50}$ determination. RuB3 showed enhanced cytotoxicity upon light irradiation in MDA-MB-231 and MCF-7 cancer cells, while a smaller light–dark difference was observed in normal H184B5F5/M10 cells. The bar chart summarizes the calculated $IC_{50}$ values under light and dark conditions, demonstrating significantly lower $IC_{50}$ values after light irradiation in cancer cells. Data are presented as mean ± SD. Statistical significance was determined by comparison between light and dark groups; ***$p < 0.001$, ns = not significant.

**Table S7.** $IC_{50}$ of **RuB3** treated with MDA-MB-231, MCF-7 and H184B5F5/M10 cells.

| | **RuB3** | | **$Ru(bpy)_3Cl_2$** | |
|---|---|---|---|---|
| Cell Line | $IC_{50}$ in Dark (μM) | $IC_{50}$ in Light (μM) | $IC_{50}$ in Dark (μM) | $IC_{50}$ in Light (μM) |
| MDA-MB-231 | 1.88 ± 0.05 | 0.50 ± 0.15 | >80 | >80 |
| MCF-7 | 1.40 ± 0.04 | 0.47 ± 0.14 | >80 | >80 |
| H184B5F5/M10 cells | 1.94 ± 0.02 | 1.64 ± 0.12 | >80 | >80 |

**Table S8. Photocytotoxicity index of RuB3 in cancer and normal cell lines.** Photocytotoxicity index (PI) values were calculated as $IC_{50}$(dark)/$IC_{50}$(light) for MDA-MB-231, MCF-7, and H184B5F5/M10 cells. Higher PI values indicate stronger light-activated cytotoxicity. RuB3 exhibited higher PI values in MDA-MB-231 and MCF-7 cancer cells than in normal H184B5F5/M10 cells, suggesting preferential photocytotoxicity toward cancer cells under light irradiation.

| Cell Line | **PI** |
|---|---|
| MDA-MB-231 | 3.76 |
| MCF-7 | 2.98 |
| H184B5F5/M10 cells | 1.18 |

**Table S9:** Comparison of $IC_{50}$ values and photocytotoxicity indices of RuB3 with representative anticancer and Ru-based compounds in MDA-MB-231 and MCF-7 cells.

| Complex | MDA-MB-231 $IC_{50}$, Light (μM) | MCF-7 $IC_{50}$, Light (μM) | PI | Reference |
|---|---|---|---|---|
| RuB3 | 0.50 ± 0.15 | 0.47 ± 0.14 | 3.76 / 2.98 | This works |
| Cisplatin | 23.4 ± 1.0 | 15.26 ± 1.24 | - | [6 7] |
| *cis*-$[Ru(bpy)_2(EtOH)_2]^{2+}$ | 25.66 ± 1.32 | 14.54 ± 2.13 | - | [6] |
| $[Re(CO)_3$(IP-TT)L]$PF_6$ | 0.0760 ± 0.0010 | - | - | [7] |
| $Ru(bpy)_2$BEDPPZ | 17.2 ± 0.9 | 74.9 ± 3.5 | - | [8] |

bpy = 2,2′-bipyridine;
IP-TT = 2-(2‘,2‘‘:5‘‘,2‘‘‘-terthiophene)-imidazo[4,5-f][1,10] phenanthroline
L = [N1-(pyridin-4-ylmethyl)-N4-(4-sulfamoylphenyl)succinamide]
BEDPPZ = benzo[e]dipyrido[3,2-a:2′,3′-c]phenazine

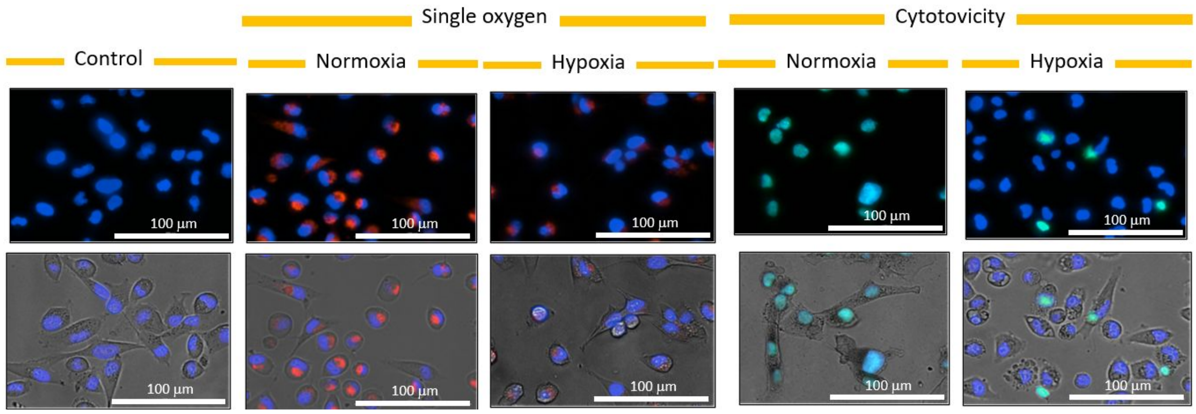


**Figure S30. Intracellular singlet oxygen generation and photocytotoxicity of RuB3 under normoxic and hypoxic conditions.** Representative fluorescence microscopy images of cells under control, normoxic, and hypoxic conditions after RuB3 treatment and light irradiation. The left panels show intracellular singlet oxygen generation, with red fluorescence indicating singlet oxygen probe activation and blue fluorescence showing DAPI-stained nuclei. The right panels show cytotoxicity staining under normoxic and hypoxic conditions, where cyan/green fluorescence indicates damaged or dead cells and blue indicates live cells. RuB3 induced clear intracellular singlet oxygen generation under normoxia, while the signal was reduced under hypoxia. Consistent with this oxygen-dependent photodynamic effect, stronger photocytotoxicity was observed under normoxic conditions than under hypoxic conditions. Scale bars: 100 μm.

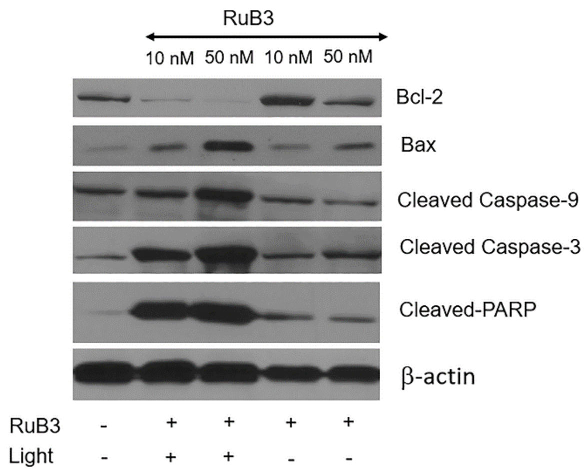


**Figure S31. RuB3 activates the mitochondria-mediated apoptotic pathway under light irradiation.** Western blot analysis of apoptosis-related proteins in cells treated with RuB3 at 10 and 50 nM under light and dark conditions. β-Actin was used as the loading control. Upon light irradiation, RuB3 decreased the anti-apoptotic protein Bcl-2 and increased the pro-apoptotic protein Bax, together with enhanced expression of cleaved caspase-9, cleaved caspase-3, and cleaved-PARP. These results indicate that RuB3-induced photocytotoxicity is associated with activation of the intrinsic apoptotic pathway.

**Table S10.** Cartesian coordination of DFT geometry-optimized of the **B3** ligand.

| Symbol | X | Y | Z |
|---|---|---|---|
| C | -6.06049 | 3.10092 | -0.69936 |
| C | -6.62922 | 4.1798 | -1.37949 |
| C | -8.01554 | 4.15175 | -1.56725 |
| C | -8.7407 | 3.06818 | -1.07363 |
| C | -6.86816 | 2.04753 | -0.24164 |
| N | -8.19918 | 2.03407 | -0.42525 |
| H | -4.99167 | 3.0917 | -0.50529 |
| H | -8.52514 | 4.96183 | -2.08318 |
| H | -9.82127 | 3.02943 | -1.20853 |
| C | -6.27399 | 0.88417 | 0.48465 |
| C | -4.9939 | 0.41794 | 0.16187 |
| C | -4.46111 | -0.69862 | 0.8275 |
| C | -5.26307 | -1.2868 | 1.8197 |
| C | -6.51911 | -0.74189 | 2.08017 |
| N | -7.03386 | 0.31145 | 1.43911 |
| H | -4.42235 | 0.88512 | -0.63366 |
| H | -4.90198 | -2.14098 | 2.38433 |
| H | -7.14789 | -1.17666 | 2.85682 |
| C | -3.1211 | -1.22653 | 0.48966 |
| C | -5.78726 | 5.32118 | -1.89503 |
| H | -4.78462 | 5.31002 | -1.4565 |
| H | -6.24803 | 6.28981 | -1.67065 |
| H | -5.67336 | 5.26257 | -2.98521 |
| C | -1.95201 | -0.40858 | 0.74763 |
| N | -0.711 | -0.8256 | 0.31358 |
| B | -0.35876 | -2.2509 | -0.19349 |
| N | -1.70074 | -2.99306 | -0.39489 |
| C | -2.97637 | -2.48885 | -0.09793 |
| C | -1.80034 | 0.83232 | 1.43296 |

| | | | |
|---|---|---|---|
| C | -0.43208 | 1.14732 | 1.3642 |
| C | 0.22272 | 0.11926 | 0.68349 |
| H | 0.03041 | 2.04071 | 1.76444 |
| C | -3.94298 | -3.45801 | -0.56621 |
| C | -1.83384 | -4.20726 | -0.97783 |
| C | -3.2044 | -4.51321 | -1.08805 |
| H | -3.60569 | -5.41638 | -1.53144 |
| C | -0.66544 | -5.01994 | -1.42478 |
| H | 0.05384 | -5.14532 | -0.60706 |
| H | -0.12735 | -4.51711 | -2.2371 |
| H | -0.99429 | -6.00414 | -1.76944 |
| C | -5.4394 | -3.3825 | -0.57758 |
| H | -5.80078 | -2.42315 | -0.96397 |
| H | -5.88126 | -3.49988 | 0.41967 |
| H | -5.84215 | -4.17774 | -1.21372 |
| C | 1.61896 | -0.05997 | 0.34581 |
| C | -2.8185 | 1.66997 | 2.15087 |
| H | -3.49202 | 1.06699 | 2.76813 |
| H | -3.45647 | 2.25268 | 1.47327 |
| H | -2.30829 | 2.38139 | 2.80955 |
| F | 0.33423 | -2.169 | -1.41366 |
| F | 0.43669 | -2.91524 | 0.74183 |
| C | 2.65584 | 0.66152 | 0.85054 |
| C | 4.05059 | 0.50314 | 0.48126 |
| H | 1.80838 | -0.8377 | -0.38631 |
| H | 2.45131 | 1.4279 | 1.5962 |
| C | 5.0293 | 1.33897 | 1.07143 |
| C | 6.37602 | 1.23174 | 0.77045 |
| C | 6.81934 | 0.2626 | -0.16072 |
| C | 5.85015 | -0.58449 | -0.76007 |
| C | 4.51346 | -0.46596 | -0.44961 |
| H | 4.70963 | 2.087 | 1.79233 |

| | | | |
|---|---|---|---|
| H | 7.08896 | 1.88752 | 1.258 |
| H | 6.1746 | -1.3353 | -1.47747 |
| H | 3.80562 | -1.1355 | -0.92666 |
| N | 8.1374 | 0.12731 | -0.50871 |
| C | 9.24272 | 0.84671 | 0.09714 |
| H | 8.37137 | -0.68738 | -1.0615 |
| C | 10.53016 | 0.59582 | -0.68395 |
| H | 9.39461 | 0.54923 | 1.14575 |
| H | 9.03133 | 1.92171 | 0.08842 |
| O | 11.55847 | 1.28726 | -0.01943 |
| H | 10.41549 | 0.94722 | -1.72347 |
| H | 10.74553 | -0.48836 | -0.7239 |
| C | 12.82727 | 1.1588 | -0.65087 |
| C | 13.845 | 1.9466 | 0.15573 |
| H | 12.77026 | 1.53671 | -1.68516 |
| H | 13.11127 | 0.09463 | -0.70612 |
| H | 13.56478 | 3.00375 | 0.20067 |
| H | 14.83603 | 1.86889 | -0.30398 |
| H | 13.90544 | 1.56242 | 1.17888 |

**Table S11.** Cartesian coordination of DFT geometry-optimized of the **RuB3** complex.

| Symbol | X | Y | Z |
|---|---|---|---|
| Ru | 4.6831 | -0.27514 | 0.02052 |
| N | 5.11952 | 0.51907 | -1.86533 |
| N | 2.60278 | -0.14473 | -0.15011 |
| N | 4.58117 | 1.78832 | 0.38138 |
| N | 4.05708 | -0.884 | 1.92101 |
| N | 4.94286 | -2.28603 | -0.47629 |
| N | 6.73182 | -0.53675 | 0.3244 |
| C | 5.07903 | 1.87319 | -1.95722 |
| C | 5.36222 | 2.51928 | -3.15757 |
| C | 5.69684 | 1.78809 | -4.29557 |
| C | 5.73605 | 0.39546 | -4.17174 |
| C | 5.44564 | -0.19491 | -2.95549 |
| C | 4.72655 | 2.57941 | -0.71199 |
| C | 4.54468 | 3.95722 | -0.62878 |
| C | 4.21276 | 4.55725 | 0.5844 |
| C | 4.08766 | 3.72126 | 1.69807 |
| C | 4.27515 | 2.35849 | 1.55816 |
| H | 4.65308 | 4.57046 | -1.51212 |
| C | 5.99334 | 2.46217 | -5.60367 |
| H | 5.18734 | 2.27021 | -6.31799 |
| H | 6.91453 | 2.07073 | -6.04076 |
| H | 6.09246 | 3.54082 | -5.48301 |
| C | 3.9788 | 6.03596 | 0.69122 |
| H | 4.31265 | 6.55886 | -0.20486 |
| H | 4.50011 | 6.45007 | 1.55676 |
| H | 2.91207 | 6.23858 | 0.82663 |
| C | 2.71838 | -0.82778 | 2.14096 |
| C | 2.16973 | -1.22853 | 3.35534 |
| C | 2.98888 | -1.6973 | 4.38146 |

| | | | |
|---|---|---|---|
| C | 4.36316 | -1.74749 | 4.12844 |
| C | 4.85394 | -1.33896 | 2.9011 |
| C | 1.91865 | -0.33034 | 1.00891 |
| C | 1.94533 | 0.2803 | -1.23996 |
| C | 0.56421 | -0.02497 | 1.09483 |
| C | -0.10502 | 0.46585 | -0.02067 |
| C | 0.59746 | 0.59354 | -1.21743 |
| H | 0.03439 | -0.13197 | 2.0302 |
| C | 7.58371 | 0.42253 | 0.72251 |
| C | 8.93776 | 0.19331 | 0.88137 |
| C | 9.46355 | -1.07653 | 0.61961 |
| C | 8.57151 | -2.06271 | 0.20399 |
| C | 7.21568 | -1.77778 | 0.06223 |
| C | 6.20869 | -2.76428 | -0.36805 |
| C | 3.96293 | -3.12106 | -0.8596 |
| C | 4.19495 | -4.45136 | -1.15554 |
| C | 5.4904 | -4.97051 | -1.05558 |
| C | 6.49798 | -4.09646 | -0.65305 |
| H | 7.14894 | 1.3929 | 0.9166 |
| H | 9.57803 | 1.00342 | 1.20636 |
| H | 2.9732 | -2.6921 | -0.92842 |
| H | 3.36728 | -5.07807 | -1.46215 |
| H | 7.51134 | -4.46079 | -0.56077 |
| H | 8.94066 | -3.05551 | -0.01129 |
| H | 1.09967 | -1.18919 | 3.5036 |
| H | 5.0531 | -2.10755 | 4.8807 |
| H | 5.91183 | -1.36702 | 2.68194 |
| H | 4.17895 | 1.68986 | 2.40168 |
| H | 3.83759 | 4.1248 | 2.67079 |
| H | 5.4653 | -1.26859 | -2.83403 |
| H | 5.3307 | 3.59834 | -3.21026 |
| H | 5.9932 | -0.23043 | -5.01664 |

| | | | |
|---|---|---|---|
| C | -1.52247 | 0.89778 | 0.08047 |
| H | 0.11199 | 0.9589 | -2.11183 |
| H | 2.53177 | 0.39043 | -2.1406 |
| C | 10.9294 | -1.35801 | 0.77998 |
| H | 11.51681 | -0.70693 | 0.12724 |
| H | 11.16551 | -2.39428 | 0.53934 |
| H | 11.24672 | -1.15867 | 1.80683 |
| C | 5.77495 | -6.41224 | -1.36313 |
| H | 5.2525 | -7.06177 | -0.65536 |
| H | 6.84136 | -6.62983 | -1.3074 |
| H | 5.41751 | -6.66929 | -2.36331 |
| C | 2.42363 | -2.11573 | 5.7076 |
| H | 1.35038 | -2.29588 | 5.64433 |
| H | 2.59169 | -1.33024 | 6.45082 |
| H | 2.91363 | -3.02039 | 6.07229 |
| C | -2.54813 | -0.00726 | -0.16276 |
| C | -1.78661 | 2.23273 | 0.42668 |
| N | -3.88414 | 0.38773 | -0.06662 |
| B | -4.36287 | 1.82562 | 0.24655 |
| N | -3.10811 | 2.68116 | 0.51583 |
| C | -3.10015 | 3.99058 | 0.86015 |
| C | -1.76837 | 4.41128 | 1.00425 |
| C | -0.92803 | 3.3292 | 0.73843 |
| H | -1.45713 | 5.40848 | 1.27667 |
| C | -4.67994 | -0.68108 | -0.33762 |
| C | -3.84157 | -1.79985 | -0.62015 |
| C | -2.52622 | -1.4027 | -0.51608 |
| H | -4.18253 | -2.79321 | -0.86548 |
| F | -5.08677 | 2.34634 | -0.84577 |
| C | -6.10708 | -0.60488 | -0.31075 |
| C | -6.91922 | -1.64959 | -0.61774 |
| C | -8.36178 | -1.66163 | -0.60668 |

| | | | |
|---|---|---|---|
| H | -6.52927 | 0.34623 | -0.01691 |
| H | -6.45872 | -2.59115 | -0.90437 |
| C | -9.04529 | -2.85136 | -0.94065 |
| C | -10.42177 | -2.92515 | -0.93806 |
| C | -11.20367 | -1.79884 | -0.59538 |
| C | -10.53082 | -0.59722 | -0.2737 |
| C | -9.15098 | -0.54052 | -0.27812 |
| H | -10.91837 | -3.85422 | -1.19544 |
| H | -8.4687 | -3.73121 | -1.20529 |
| H | -11.09615 | 0.29045 | -0.02528 |
| H | -8.67536 | 0.3998 | -0.02703 |
| N | -12.5663 | -1.90318 | -0.56047 |
| C | -13.46032 | -0.77043 | -0.40302 |
| C | -14.88976 | -1.25809 | -0.22704 |
| H | -13.41389 | -0.08864 | -1.26262 |
| H | -13.17899 | -0.20042 | 0.48571 |
| O | -15.70924 | -0.11624 | -0.0776 |
| H | -14.9577 | -1.90891 | 0.65597 |
| H | -15.2041 | -1.8456 | -1.10309 |
| C | -17.08546 | -0.43973 | 0.10531 |
| C | -17.86508 | 0.85173 | 0.25306 |
| H | -17.20081 | -1.06999 | 0.99821 |
| H | -17.44662 | -1.01717 | -0.75731 |
| H | -12.9555 | -2.71866 | -1.01075 |
| H | -17.50692 | 1.41874 | 1.1155 |
| H | -18.92718 | 0.6391 | 0.39543 |
| H | -17.75271 | 1.47112 | -0.63985 |
| F | -5.204 | 1.82335 | 1.37911 |
| C | -4.34586 | 4.78751 | 1.03751 |
| H | -4.94482 | 4.77476 | 0.12356 |
| H | -4.10153 | 5.81882 | 1.28939 |
| H | -4.96662 | 4.36308 | 1.83029 |

| | | | |
|---|---|---|---|
| C | 0.56908 | 3.38043 | 0.8004 |
| H | 0.88894 | 4.38724 | 1.07091 |
| H | 1.0349 | 3.12724 | -0.15441 |
| H | 0.97364 | 2.69166 | 1.54522 |
| C | -1.33796 | -2.28745 | -0.73173 |
| H | -1.66946 | -3.29204 | -0.99618 |
| H | -0.71762 | -2.35905 | 0.16453 |
| H | -0.69776 | -1.91756 | -1.53563 |

## References


1. Paul, S.; Kundu, P.; Kondaiah, P.; Chakravarty, A. R., BODIPY-Ruthenium(II) Bis-Terpyridine Complexes for Cellular Imaging and Type-I/-II Photodynamic Therapy. *Inorg. Chem.* **2021,** *60* (21), 16178-16193.
2. Zhou, Q.-X.; Lei, W.-H.; Hou, Y.-J.; Chen, Y.-J.; Li, C.; Zhang, B.-W.; Wang, X.-S., BODIPY-Modified Ru(II) Arene Complex—A New Ligand Dissociation Mechanism and a Novel Strategy to Red-Shift the Photoactivation Wavelength of Anticancer Metallodrugs. *Dalton Trans.* **2013,** *42* (8), 2786-2791.
3. Yu, X.; Gao, F.; Zhao, W.; Lai, H.; Wei, L.; Yang, C.; Wu, W., BODIPY-Conjugated Bis-Terpyridine Ru(II) Complexes Showing Ultra-Long Luminescence Lifetimes and Applications to Triplet–Triplet Annihilation Upconversion. *Dalton Trans.* **2022,** *51* (24), 9314-9322.
4. Paul, S.; Sahoo, S.; Sahoo, S.; Jayabaskaran, C.; Chakravarty, A. R., Bichromophoric BODIPY and Biotin Tagged Terpyridyl Ruthenium(II) Complexes for Cellular Imaging and Photodynamic Therapy. *Eur. J. Inorg. Chem.* **2022,** *2022* (34), e202200487.
5. Aksoy, Burcu T.; Özcan, E.; Bulut, O.; Kazan, Hasan H.; Çoşut, B., Synthesis, Photophysical Properties, and Photodynamic Therapy Efficacies of Meso-Pyridine BODIPYs and Their Ruthenium Complexes. *Appl. Organomet. Chem.* **2025,** *39* (12), e70459.
6. Yang, Y.; Gao, Y.; Sun, Y.; Zhao, J.; Gou, S., Study on the Multimodal Anticancer Mechanism of Ru(II)/Ir(III) Complexes Bearing a Poly(ADP-ribose) Polymerase 1 Inhibitor. *J. Med. Chem.* **2023,** *66* (19), 13731-13745.
7. Su, X.; Wang, W.-J.; Cao, Q.; Zhang, H.; Liu, B.; Ling, Y.; Zhou, X.; Mao, Z.-W., A Carbonic Anhydrase IX (CAIX)-Anchored Rhenium(I) Photosensitizer Evokes Pyroptosis for Enhanced Anti-Tumor Immunity. *Angew. Chem.* **2022,** *134* (8), e202115800.
8. Zhao, X.; Li, L.; Yu, G.; Zhang, S.; Li, Y.; Wu, Q.; Huang, X.; Mei, W., Nucleus-enriched Ruthenium Polypyridine Complex Acts as a Potent Inhibitor to Suppress Triple-negative Breast Cancer Metastasis In vivo. *Comput. Struct. Biotechnol. J.* **2019,** *17*, 21-30.
